# Genome-wide identification, and characterization of the CDPK gene family reveal their involvement in abiotic stress response in *Fragaria x ananassa*

**DOI:** 10.1038/s41598-020-67957-9

**Published:** 2020-07-06

**Authors:** Rosane Lopes Crizel, Ellen Cristina Perin, Isabel Lopes Vighi, Rafael Woloski, Amilton Seixas, Luciano da Silva Pinto, César Valmor Rombaldi, Vanessa Galli

**Affiliations:** 1 Departamento de Ciência e Tecnologia Agroindustrial, Universidade Federal de Pelotas, Pelotas, Brasil; 2 Programa de Pós-Graduação em Tecnologia de Processos Químicos e Bioquímicos, Universidade Tecnologia Federal do Paraná, Pato Branco, Brasil; 3 Centro de Desenvolvimento Tecnológico, Universidade Federal de Pelotas, Pelotas, Brasil

**Keywords:** Genetics, Molecular biology, Plant sciences

## Abstract

Calcium-dependent protein kinases (CDPKs) are encoded by a large gene family and play important roles against biotic and abiotic stresses and in plant growth and development. To date, little is known about the CDPK genes in strawberry (*Fragaria x ananassa*). In this study, analysis of *Fragaria x ananassa* CDPK gene family was performed, including gene structures, phylogeny, interactome and expression profiles. Nine new CDPK genes in *Fragaria x ananassa* were identified based on RNA-seq data. These identified strawberry *FaCDPK* genes were classified into four main groups, based on the phylogenetic analysis and structural features. *FaCDPK* genes were differentially expressed during fruit development and ripening, as well as in response to abiotic stress (salt and drought), and hormone (abscisic acid) treatment. In addition, the interaction network analysis pointed out proteins involved in the ABA-dependent response to plant stress via Ca^2+^ signaling, especially RBOHs. To our knowledge, this is the first report on CDPK families in *Fragaria x ananassa*, and it will provide valuable information for development of biofortified fruits and stress tolerant plants.

## Introduction

Plants are constantly exposed to stress conditions, such as drought, low or high temperature, and high salinity^1^. Plants adapt to these conditions by capturing external signals and modulating their responses using complex mechanisms. These mechanisms involve the perception of the stimulus and subsequent signal transduction, which lead to the activation of various chemical, molecular, biochemical, and physiologic changes, improving plant plasticity^2^. The calcium ion (Ca^2+^) plays central roles in the regulation of different physiological processes in plants as an important secondary messenger. Transient changes in the cytoplasmic concentration of Ca^2+^ are detected by several types of sensor proteins that initiate rapid signal transduction processes by triggering cascades of phosphorylation events. Several major classes of Ca^2+^ binding proteins have been characterized in plants, including Ca^2+^-dependent protein kinases (CDPK), calmodulin (CaM), CAM-like proteins (CML), and calcineurin B-like proteins (CBL)^3–6^. Among these, CDPK constitutes a large calcium-sensing family only found in plants, protists, oomycetes, and green algae, and not in animals and fungi^7^.

CDPK structures include four domains: N-terminal, self-regulatory/autoinhibitory, serine/threonine kinase, and finally calmodulin-like regulatory domains (CaM-LD)^8^. The CaM-LD domain contains three or four EF-hand Ca^2+^-binding motifs that recognize distinct Ca^2+^ signatures with variable affinities. The C-terminal lobe of the CaM-LD binds Ca^2+^ with high affinity. At low Ca^2+^ levels, the structure is stabilized by the interaction of this lobe with the auto-inhibitory region of the protein. Otherwise, a conformational change induced by the binding of Ca^2+^ to the low-affinity N-terminal lobe of CaM-LD results in the release of the auto-inhibition^9,10^.

CDPKs are involved in stress signaling, hormone response, and metabolic pathway regulation^7^. As an example, *Os*CDPK4 was reported to play important roles in salt tolerance and drought stress in rice^11^. *Os*CDPK4 also protects against the plant-pathogenic fungus *Magnaporthe oryzae* by regulating the immune system of plants^12^. Silencing and overexpression studies of *OsCPK9* plays a positive role in drought stress tolerance^13^*.* In Arabidopsis, *AtCPK8* was reported to interact with and regulate the activity of Catalase-3, and *cpk8* mutants showed impaired abscisic acid (ABA), H_2_O_2_, and Ca^2+^ induced stomatal closing^14^. Moreover, CDPKs have been associated with the regulation of the secondary metabolite production^15^. Resveratrol content was increased in transgenic plant *Vitis amurensis* cells overexpressing *VaCPK20* or *Va*CPK29^16^. One of the mechanisms that CDPK uses to regulate the metabolism of phenylpropanoids is through phosphorylation of the PAL enzyme (phenylalanine ammonium lyase), the first enzyme in the metabolic pathway of phenylpropanoids^17^.

The CDPKs are encoded by multigenic families: 31 genes of CDPKs were identified in rice (*Oryza sativa*)^18,19^; 34 genes in *Arabidopsis thaliana*^20^; 29 genes in tomato (*Solanum lycopersicum*)^21^; 25 genes in canola (*Brassica napus*)^22^; and 29 genes in Poplar (*Populus trichocarpa)*^7^; among others. In *Fragaria* × *ananassa*, however, only two *CPDK* gene sequences have been identified to date, and the roles of these genes/enzymes in stress signaling, fruit ripening, and tissue-specific gene expression remain uncertain ^23,24^.

As an economically important fruit species, the strawberry octoploid (*Fragaria x ananassa*) is cultivated and consumed around the world due to its superior sensorial and nutritional characteristics^25^*.* Strawberry fruit contains high levels of vitamin, mineral, anthocyanins and phenolic compounds, and is therefore considered an antioxidant source. Our research group recently assembled and annotated the transcriptome of strawberry fruits submitted to saline stress and water deficit ^26^. Here, we identified nine new *CDPK* genes in *Fragaria x ananassa* through a deep search of this transcriptome. Our analyses yielded detailed information on these *FaCDPKs*, including phylogenetic trees and genomic structures. Expression profiles of *FaCDPK* genes with respect to salt and drought stress responses as well as during fruit development and ripening, providing directions for further studies and biotechnological applications.

## Results

### Identification of CDPK genes in strawberry

Bioinformatics methods were used to identify CDPK family genes in the strawberry (*F. ananassa*) genome. Genomic analysis was performed using the RNA-seq data from the Sequence Read Archive (SRA) (accession code SRP148865). The presence of typical CDPK domains, including the Ca^2+^ binding, kinase, N-terminal, and autoinhibitory domains, were verified using the PROSITE tools to verify the reliability of candidate sequences. Using this procedure, nine possible new CDPK sequences in *F. ananassa* were identified and designated as *FaCDPK3* to *FaCDPK11* according to the proposed CDPK gene nomenclature (*FaCDPK1* and *FaCDPK2* sequences were previously described)^19^. All identified *FaCDPK* genes encoded proteins with amino acid numbers and molecular weights from 255 to 600 and from 28.7 to 67.1 kDa, respectively. The isoelectric point varied between 4.51 (*FaCDPK9*) and 9.09 (*FaCDPK2*). All identified sequences corresponded to the complete protein sequence and featured at least one kinase domain and one EF-hand domain as shown in Table 1. Five sequences included predicted myristoylation motifs at their N-terminus sites, and six contained at least one palmitoylation site, an indication of possible protein-membrane interaction. Bioinformatics prediction of subcellular protein localization also suggested that different members can localize into different subcellular compartments such as the chloroplast, cell membrane, nucleus, and cytoplasm (Table S1).

**Table 1:** Information on the sequences of calcium-dependent protein kinase (CDPK) identified in strawberry (*Fragaria x ananassa*).

**Gene name**	**No. of amino acid**	**Kinase domain**	**EF-hand**	**pI**	**M.W. (kDa)**	**Myristoylaton motif**	**Palmitoylation prediction**
FaCDPK1	255	1	2	7.13	28.7	No	Yes
FaCDPK2	552	1	4	9.09	62.6	Yes	No
FaCDPK3	451	1	1	9.57	51.0	Yes	No
FaCDPK4	541	1	4	6.53	51.1	Yes	Yes
FaCDPK5	562	1	4	5.44	62.5	No	Yes
FaCDPK6	547	1	4	6.62	62.1	No	Yes
FaCDPK7	545	1	4	6.15	61.2	Yes	Yes
FaCDPK8	600	1	1	8.93	67.1	Yes	Yes
FaCDPK9	351	1	4	4.51	39.3	No	No
FaCDPK10	519	1	4	6.02	58.3	No	No
FaCDPK11	544	1	4	6.06	61.3	Yes	Yes

### Gene structure and phylogenetic analysis

**Figure 1:**
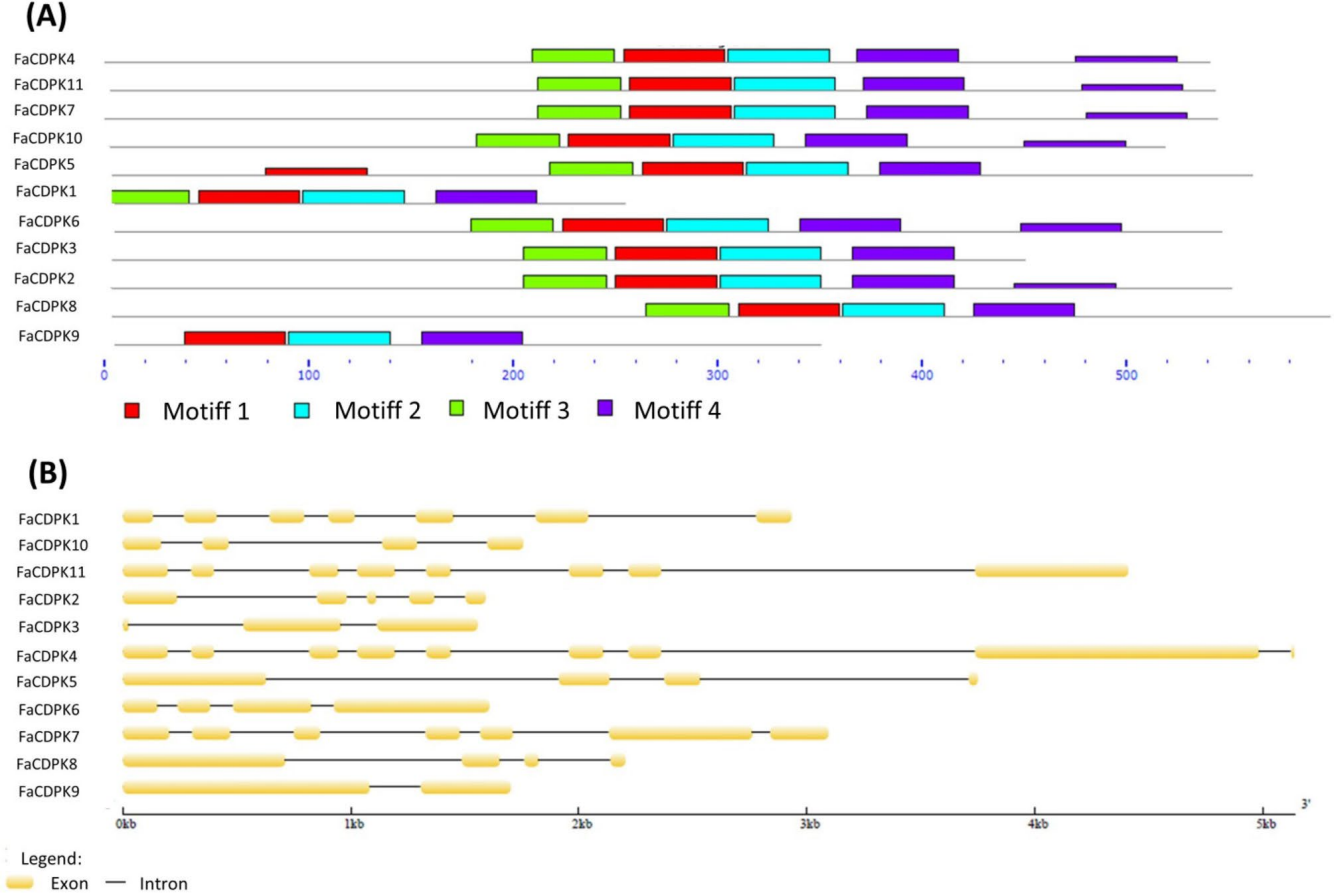
(**A**) Organization of the different motifs in 11 calcium-dependent protein kinases (CDPK) genes present in *Fragaria x ananassa*. Preserved motifs were detected using the online MEME tools (https://meme.sdsc.edu/meme/intro.html). (**B**) Structure of the exons-introns in 11 calcium-dependent protein kinases (CDPK) genes present in *Fragaria x ananassa.* Exons and Introns are indicated by orange boxes and black lines, respectively.

Structural divergence among the *FaCDPKs*, such as the presence/absence and positions of domains, may provide information regarding the evolutionary history of this gene family^27^. Therefore, eleven identified *FaCDPK* genes were analyzed to confirm the presence of typical CDPK domains. Four conserved motifs were identified (Fig. 1A). Six sequences included one motif (Motif 4) in two different regions, however with different block heights. The height of a block indicates the significance of the match (taller blocks are more statistically significant). In addition, one sequence included only three of the four conserved motifs, suggesting the occurrence of duplication and deletion events from the ancestral sequence that characterize modifications including domain deletion and insertion.

The organization of the exons/introns and the number of introns present may also indicate the evolutionary history within a gene family^28^. An analysis of the structures of the eleven *FaCDPKs* revealed that four *FaCDPKs* (*FaCDPK10*, *5*, *6,* and *8*) include three introns, two *FaCDPKs* (*FaCDPK1* and *7*) include six introns, *FaCDPK4* and *FaCDPK11* include seven introns. *FaCDPK9*, *FaCDPK3* and *FaCDPK2* included one, two, and three introns, respectively (Fig. 1B).

**Figure 2:**
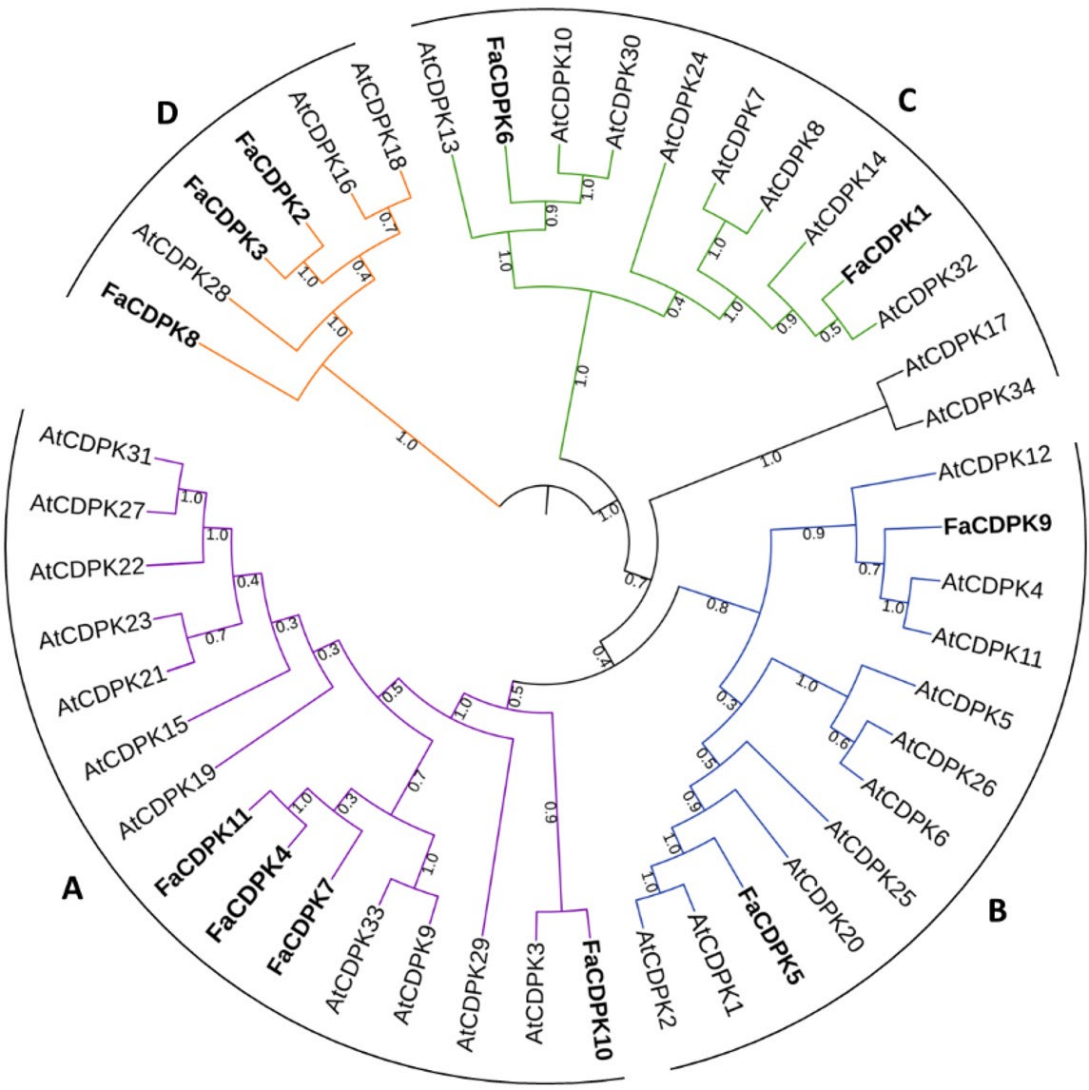
Phylogenetic relationship between the calcium dependent protein kinases (CDPK) of strawberry (*Fragaria x ananassa*) and *Arabidopsis thaliana*. The phylogenetic tree was constructed based on an amino acid sequence alignment using Neighbor-Joining method with *p*-distance and bootstrap analysis (1,000 replicates).

The evolutionary relationships of CDPK genes were also observed through phylogenetic trees. The phylogenetic trees were constructed using the Neighbor Joining grouping method with different substitutions models. The five trees obtained showed similar topology. The tree obtained with the substitution model *p*-distance is presented in Fig. 2, and the others as Figure S1. The eleven *FaCDPK s*were assigned to four main subfamilies according to tree topology (Groups A, B, C and D), which matched the *At*CDPKs classification (Fig. 2). Eleven *AtCDPKs* and four *FaCDPKs* (*FaCDPK7*, *4*, *11* and *10*) belong to group A; twelve *AtCDPKs* and two *FaCDPKs* (*FaCDPK9* and *5*) to group B; eight *AtCDPKs* and two *FaCDPKs* (*FaCDPK6* and *1*) to group C; and three *AtCDPKs* and three *FaCDPKs* (*FaCDPK8*, *3* and *2*) to group D. The results of the phylogenetic analysis of the predicted *FaCDPK* protein sequences revealed that there is no egalitarian representation of *F. ananassa* and *A. thaliana* proteins in the four subgroups, since the ancestral CDPK gene was subjected to multiple gene duplication events.

### Chromosomal distribution and synteny analysis of CDPK genes

**Figure 3:**
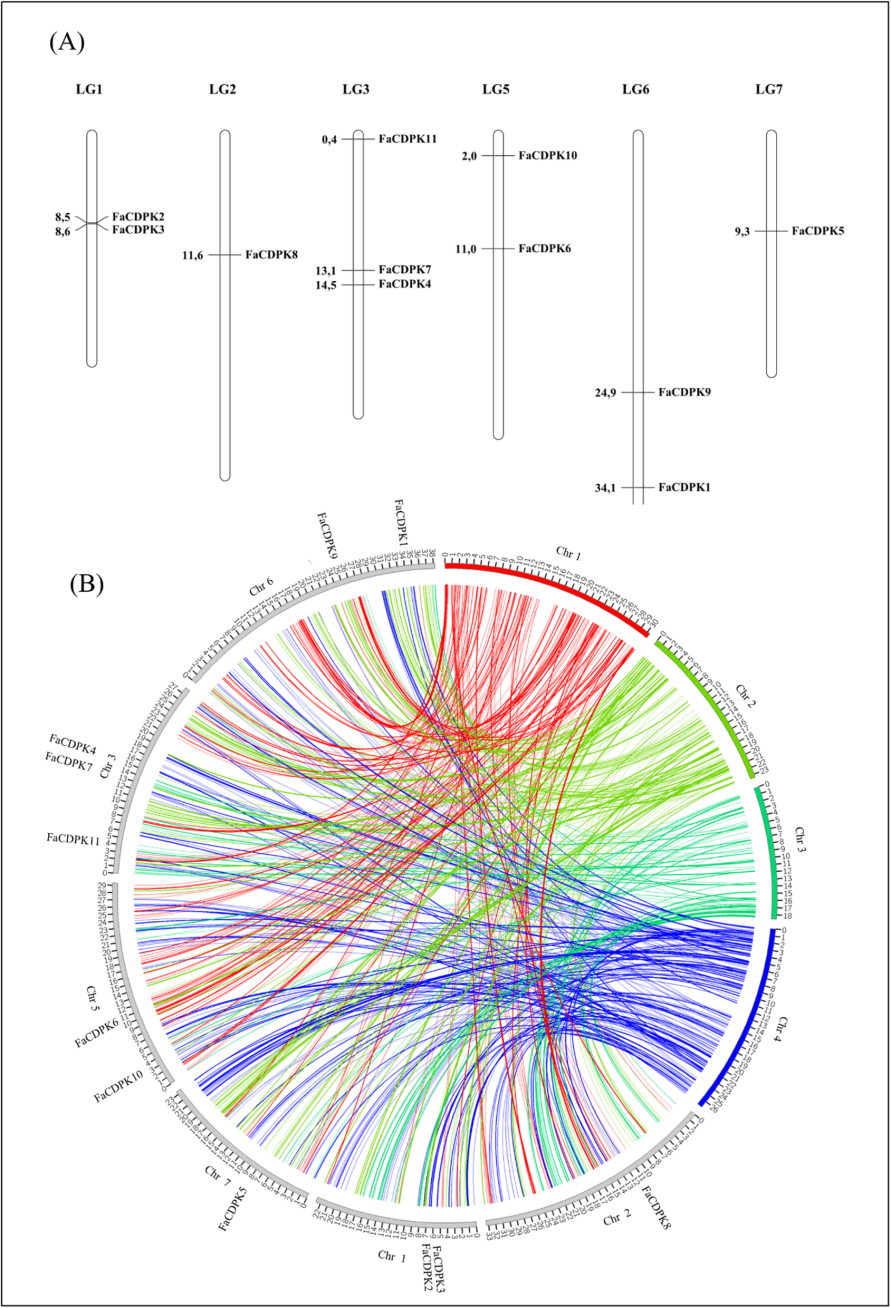
(**A**) Chromosomal distribution of the *FaCDPK* genes in the chromosomes of *F. vesca*. Chromosome numbers are provided at the top of each chromosome. (**B**) Synteny analysis of CDPK genes between strawberry (*Fragaria x ananassa*) and *Arabidopsis thaliana*.

The *CDPK* genes were mapped across the chromosomes of the wild-type strawberry genome (*Fragaria vesca*). Chromosome 4 was the only chromosome with no *FaCDPK* gene (Fig. 3A). On the other hand, chromosome 3 included three *FaCDPKs* (*FaCDPK4*, *FaCDPK7,* and *FaCDPK11*), chromosomes 1, 5, and 6 included two *FaCDPKs* each (*FaCDPK2* and *FaCDPK3*, *FaCDPK6* and *FaCDPK10*, *FaCDPK1,* and *FaCDPK9*, respectively), and chromosomes 2 and 7 included only *FaCDPK8* and *FaCDPK5* genes.

Synteny analysis was carried out to investigate the overall structural variation within the genome, such as fission and fusions of chromosomes. *FaCDPKs* were found to maintain the synteny with *CDPKs* from the four *Arabidopsis* chromosomes (Fig. 3B). Two genes linked to each other by one line in Fig. 3B are defined as syntenic genes. Individual chromosomes from the two genomes were mostly connected by lines of the same color, indicating an evolutionary similarity between these genomes. Most of the CDPK genes were preserved during polyploidization, indicating their evolutionary importance. CDPK genes were also evenly-distributed within these genomes, and synteny analysis further indicated that the syntenic CDPK gene pairs were widely-distributed within the genomes.

Evolutionary pattern prediction based on the calculation of synonymous (Ks) and non-synonymous (Ka) substitution rates provides information regarding the type of gene pair selection during the divergence process, such as purifying, positive, and neutral selection^29^. Ka/Ks ratios below, equal to, and above 1.00 indicate purifying, neutral, and positive selection types^30^. Here, the Ka, Ks, and Ka/Ks of the orthologous gene pairs were calculated based on the phylogenetic tree analysis results (Table S2). Time between 22 orthologous pairs of *FaCDPK* genes was also determined. Interestingly, 12 out of 22 orthologous *FaCDPK* gene pairs yielded a Ka/Ks ratio < 1.00, indicating purifying selection. However, the remaining 10 *FaCDPK* genes exhibited a Ka/Ks ratio > 1.00, which suggests a positive selection of the orthologous pairs. Furthermore, the divergence time and duplication time ranged from 0.07 to 8.4 (Ks values) and 2.4 to 280 million of years ago (MYA), respectively.

### Expression of *FaCDPK* genes during the development of the *F. ananassa*

**Figure 4:**
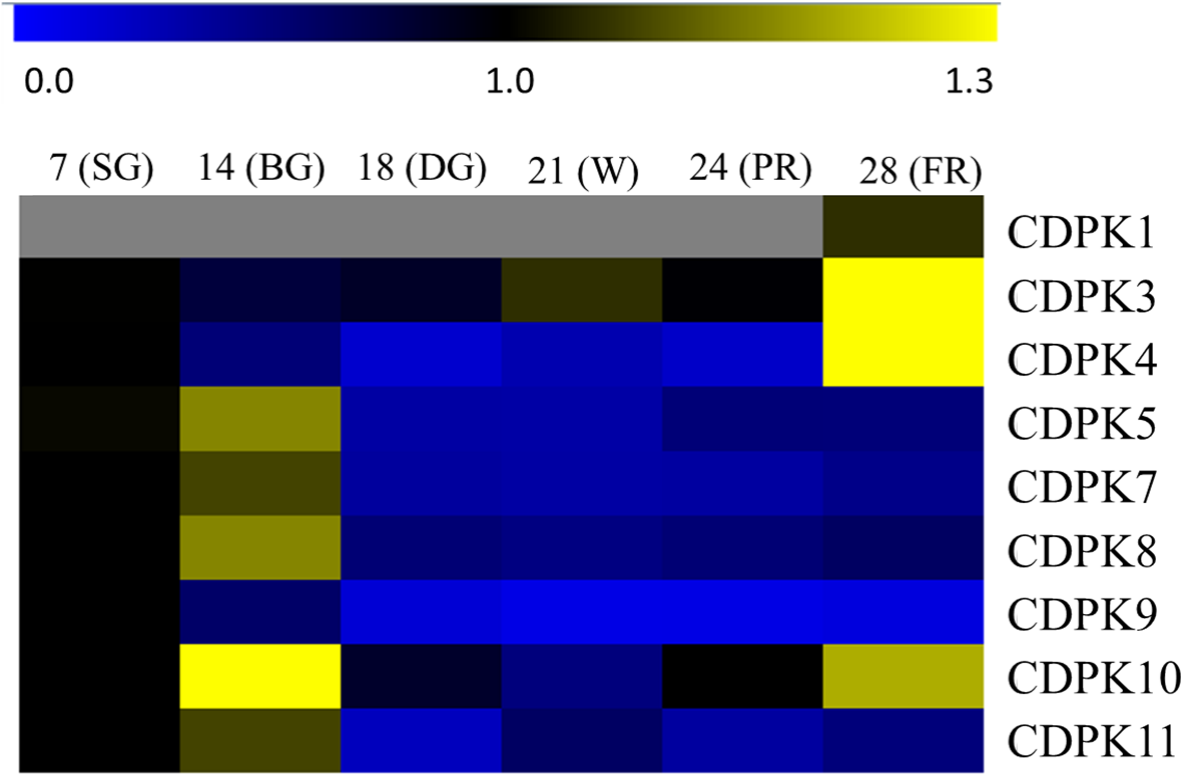
Expression of calcium-dependent protein kinases (CDPK) during fruit development in *Fragaria* × *ananassa.* The values of relative expression levels according to the RT-qPCR assays were log-transformed to perform heatmaps. The yellow and blue colors represent high and low values of relative expression, respectively. Grey color indicates not detected. The expression levels of PIRUV_DESCARB, DBP and HISTH4 were used as reference gene to normalize the expression. The data was performed with four biological and three technical replicates. Developmental stages correspond to 7 (small green, SG), 14 (big green, BG), 18 (degreening DG), 21 (white, W), 24 (partial red, PR), and 28 (full red, FR) days after anthesis.

In order to elucidate the regulatory roles of *FaCDPKs* during the development and ripening of strawberry fruits, RT-qPCR expression analysis was performed. Highest accumulation of *FaCDPK3*, *FaCDPK4,* and *FaCDPK10* transcripts was found in the final maturation stage (FR), whereas for the other genes the accumulation occurred in the initial stages (SG or BG) (Fig. 4). Only*FaCDPK3* showed increased expression during fruit maturation, and no *FaCDPK1*expression was detected in young fruits.

### Expression of *FaCDPK* genes under abiotic stress conditions

**Figure 5:**
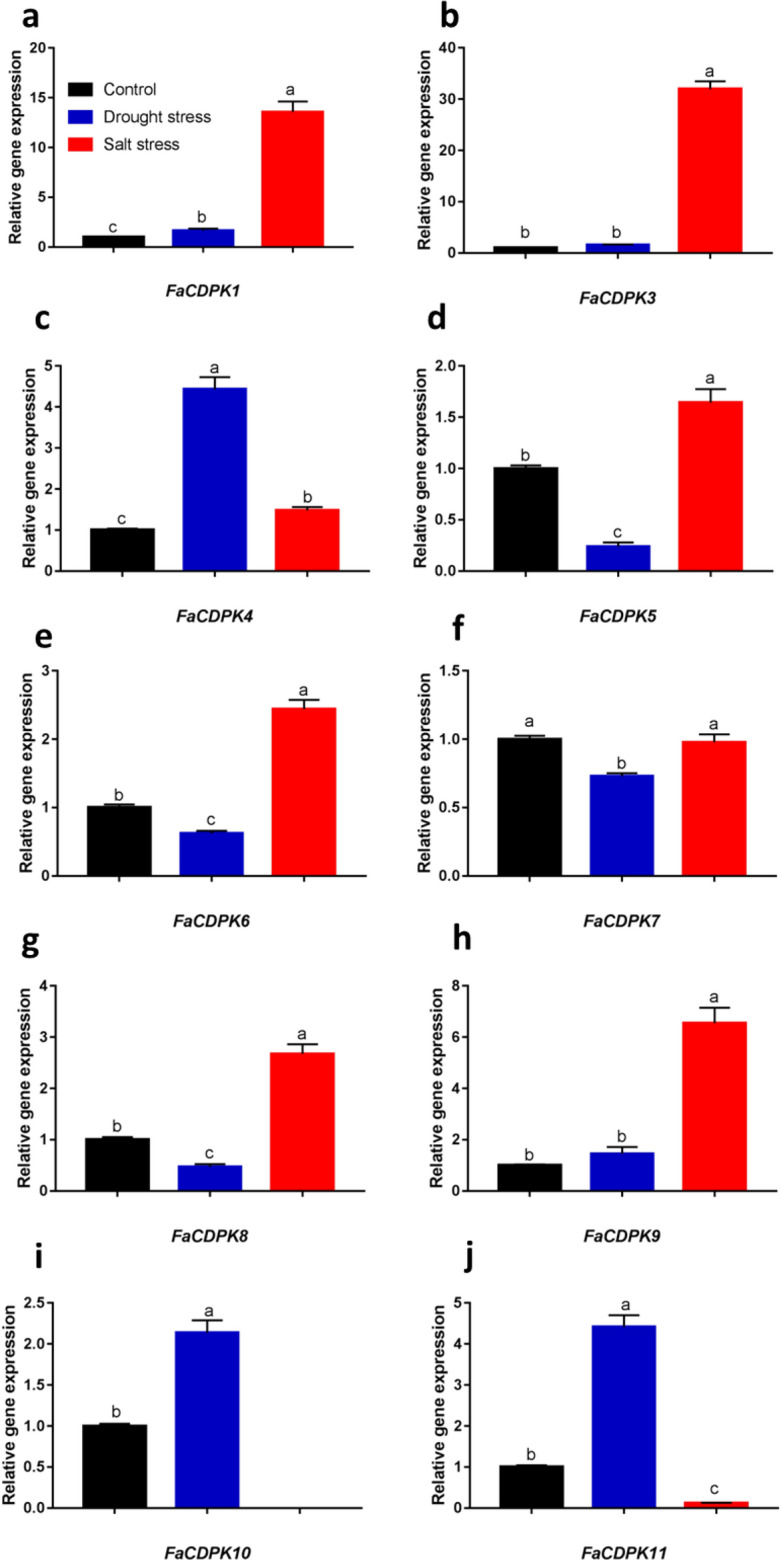
Expression of calcium-dependent protein kinases (CDPK) under salt and drought stress treatment in *Fragaria* × *ananassa*. The expression level of PIRUV_DESCARB, DBP and HISTH4 was used as reference gene to normalize each reaction. The data was from four biological and three technical replicates. Data were subjected to one-way ANOVA test and when significant (p ≤ 0.05) were submitted to a comparison of means by Tukey test at 5% of probability. Different letters indicate significant differences between treatment.

CDPKs have been reported as essential factors in regulating plant tolerance to biotic and abiotic stresses. The gene expression profiles of *FaCDPK*s under salt and drought stress conditions were evaluated (Fig. 5). The expression of nine *Fa*CDPKs was altered in response to stress treatments, and the expression levels of six these genes (*FaCDPK1*, *FaCDPK3*, *FaCDPK5*, *FaCDPK6*, *Fa*C*DPK8,* and *FaCDPK9*) increased upon salt treatment, whereas three genes (*FaCDPK4*, *FaCDPK10* and *FaCDPK11*) were overexpressed under drought conditions. The highest differences were observed for *FaCDPK1* and *FaCDPK3*, which showed 15- and 30-fold higher expression levels in salt and drought stress than control fruit, respectively. In contrast, lower expressions of *FaCDPK5*, *FaCDPK6*, *FaCDPK7* and *FaCDPK8* were detected under drought stress. This suggests that different genes are involved in responses to salt and drought stresses.

**Figure 6:**
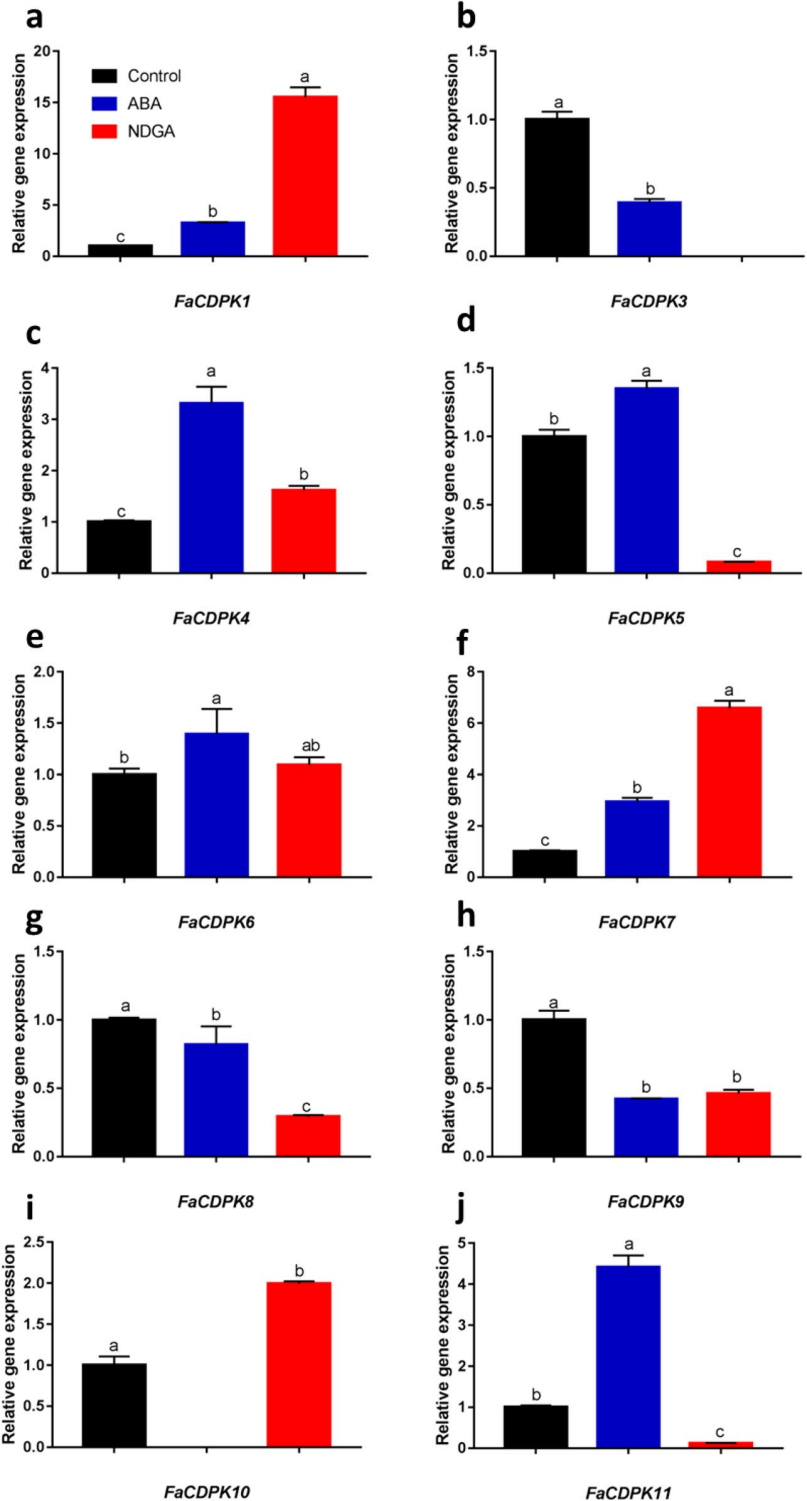
Expression of calcium-dependent protein kinases (CDPK) under abscisic acid (ABA) and nordihydroguaiaretic acid (NDGA) treatment in *Fragaria* × *ananassa*. The expression level of PIRUV_DESCARB, DBP and HISTH4 was used as reference gene to normalize each reaction. The data was from four biological and three technical replicates. Data were subjected to one-way ANOVA test and when significant (p ≤ 0.05) were submitted to a comparison of means by Tukey test at 5% of probability. Different letters indicate significant differences between treatment.

The regulation of tolerance to biotic and abiotic stresses is mediated by CDPKs. In some cases, this is accompanied by the modulation of ABA signaling. In order to better understand this relationship, the expression of *FaCDPK* transcripts was evaluated in strawberry fruits treated with ABA and an ABA synthesis inhibitor (NDGA). The expression of *FaCDPK4* and *FaCDPK11* genes increased upon ABA treatment (Fig. 6). The expression of the *FaCDPK1* and *FaCDPK7* genes was also improved by ABA and NDGA treatment, however the highest accumulation of transcripts of these genes was observed in fruits treated with NDGA (*FaCDPK1* showed 15-fold higher expression than that of control fruits).

### Protein–protein interaction network analysis

**Figure 7:**
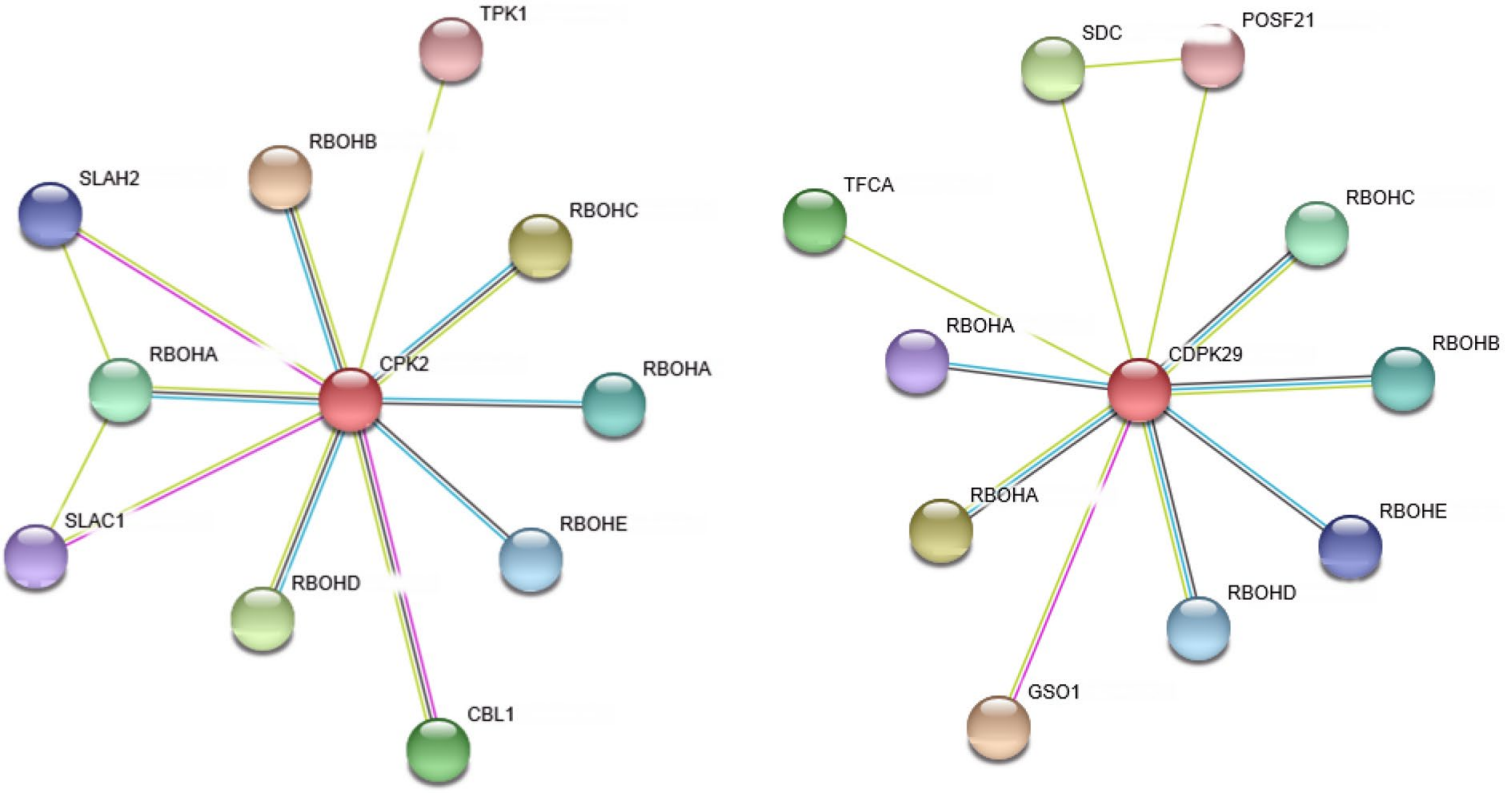
Interaction network analysis of *Fa*CDPK4 and *Fa*CDPK11 proteins identified in *Fragaria x ananassa*. The analysis was performed with the respective homologous in *Fragaria x vesca* (CPK2 for *FaCDPK4* and CDPK29 for *FaCDPK11*). Line color indicates the type of interaction evidence: Black line indicates co-expression; Green line indicates textmining; Blue line indicates databases.

The above results indicate a possible involvement of *FaCDPK4* and *FaCDPK11* genes in the abiotic stress response through interactions between CDPK and other proteins. Network interaction analysis is a powerful method to study the gene function^31^. An interaction network for CDPK proteins was constructed based on the orthologous gene of *Fragaria x vesca* using STRING 10 database tools. Accordingly, CDPKs were found to be involved in signaling pathways related to plant growth and development and abiotic stress response. This analysis showed that *Fv*CPK2 (homologous to *FaCDPK4* from *Fragaria x ananassa*) is associated with several respiratory oxidase homolog proteins (RBOH), which promote ROS scavenging (Fig. 7). *Fv*CPK2 also interacts with calcineurin B-like protein (CBL)—a positive regulator of salt and drought responses- as well as thiamine pyrophosphokinase (TPK) and guard cell S-type anion channel (SLAC), which are involved in energy production and stomatal responses. Similarly, *Fv*CDPK29 (homologous to*FaCDPK11*) interacts with RBOH proteins. In addition, *Fv*CDPK29 interacts with serine decarboxylase (SDC), an enzyme related to the formation of membrane phospholipids and probable transcription factor (POSF), a transcription factor gene. *Fv*CDPK29also interacts with LRR receptor-like serine/threonine-protein kinase (GSO), a positive cell division regulator and tubulin-folding cofactor (TFCA), which play roles in modulating tubulin folding. Thus, network analysis results indicate a possible involvement of *Fv*CDPK29 in the maintenance of cell integrity. Given the homology between these CDPKs to *FaCDPK4* and *FaCDPK11*, it is possible that these *Fa*CDPKs play similar roles.

## Discussion

The first calcium-dependent protein kinase (CDPK) was identified over 30 years ago in pea^32^. CDPKs have since been identified in several plants and some protozoa^20^. In *Arabidopsis thaliana* and *Oryza sativa* (rice), 34 and 31 genes of this family have been identified, respectively^18,20^. However, only two CDPK-coding sequences were identified in strawberry (*F. ananassa*) thus far. Here, we analyzed genomic and transcriptomic data from *F. ananassa*, and revealed the presence of nine novel CDPK genes. Due to the scarcity of DNA and RNA sequence data for this species, further *CPDK* variants other than those described here are likely to be identified as more sequencing data are made available in sequence databases.

CDPK structure contains at least one variable N-terminal domain, a protein kinase domain, a junction domain, a calmodulin-like Ca^2+^ binding domain and a C-terminal domain. All CDPKs analyzed here included complete sequences, including the N variable domain, the C terminal domain, at least one kinase domain for target phosphorylation, and one EF-hand domain. However, seven of the nine novel sequences exhibited a pattern of four EF-hand motifs, which allow Ca^2+^ binding as previously reported for Arabidopsis, rice and wheat CDPKs^4^. This configuration, featuring a tandem of EF-hand domains able to bind up to four calcium ions, possibly originated from a single motif via two cycles of gene duplication and fusion, and enabled cooperativity between the calcium binding sites. From an evolutionary point of view, this structural organization in the form off our repetitive structures may be interpreted as a way to obtain different domain movements, and thereby diversify molecular recognition^33^. The EF hand motifs mostly exist in pairs to increase structural stability. The presence of a single EF-hand motif, as observed in *FaCDPK3* and *FaCDPK8*, was reported to drastically reduce the sensitivity of the kinase for calcium-binding, and the subsequent calcium-induced conformational change^6^. For example, a 60-fold reduction in the binding affinity for Ca^2+^ ions was demonstrated for a CDPK with a single EF-hand motif in *Plasmodium falciparum*^34^. Taking into account the wide variety of CDPK genes in plants, investigation of the factors determining the isoforms responding to different signals, the effects on protein phosphorylation and physiological responses is important. The signaling specificity may be determined by the subcellular location of the CDPK proteins, which can be regulated at translational and post-translational levels. In *silico* predictions indicate that *Fa*CDPKs are localized in several cell compartments, including the chloroplast, nucleus, peroxisome and cytoplasm. Most of *Fa*CDPKs were also predicted to include N-myristoylation and/or palmitoylation motifs. CDPK genes which have a N-myristoylation motif tend to localize in the plasma membranes^35,36^. This myristoylation may function as part of a primary signaling process that directs these proteins to a cell membrane binding site. In addition, palmitoylation, another type of lipid modification, was also reported to be necessary for stability of membrane association^35^. N-myristoylation and palmitoylation motifs were observed in five and six of the *Fa*CDPKs evaluated in our study, respectively. However, the association of CDPKs with the membrane is complex, and may be affected by the presence of other motifs. For example, in wheat (*Triticum aestivum* L), *TaCPK3* and *TaCPK15* without myristoylation regions were associated with membranes^37^, whereas in maize (*Zea mays*) *ZmCPK1* with an N-myristoylation pattern, was found in the cytoplasm and nucleus^38^. Other studies have shown that the subcellular locations of CDPKs can be restricted to a single compartment or widely-distributed throughout the cell. CDPKs have already been observed in the plasma membrane, cytoplasm, nucleus, endoplasmic reticulum, mitochondria, chloroplasts, oily bodies, peroxisomes and the Golgi complex^39^. Therefore, additional in vitro studies are necessary to determine the subcellular localization of *Fa*CDPKs.

Genes originating from a common ancestor share similar protein function. Thus, a syntenic analysis was performed, and a phylogenetic tree was constructed in order to understand the evolutionary relations between the CDPKs present in *F. ananassa* and *A. thaliana*. All identified *FaCDPK* homologs in *Arabidopsis* showed high sequence similarity, indicating a high level of conservation and a common ancestor for each potential orthologous pair. Four groups (A, B, C, and D), each with at least one counterpart from Arabidopsis, were identified in the phylogenetic tree. This finding implies a functional conservation of CDPKs between the two plants. Similar evolutionary classification was also found in other species, such as grape, pineapple, cassava, and rice^40–43^. Here, *FaCDPK4*, *FaCDPK7,* and *FaCDPK11* were present with *AtCDPK23* in subgroup A. *AtCDPK23* plays important roles in drought and saline stress responses^44^, suggesting possible involvement of *FaCDPK4* and *FaCDPK11* in response to these stress conditions in strawberry. *GrCDPK16* has been reported to be in the same phylogenetic group as *At*CDPK23 and associated with plant tolerance to drought stress in cotton^45^. *FaCDPK6* shares 92% sequence identity with *AtCDPK30*. Hence, *FaCDPK6* may be involved in hormonal signaling pathways in strawberry. *CDPK* genes are also involved in resistance to different pathogens. In potato, *StCDPK7* was reported to promote resistance against *Phytophthora infestans* infection^46^. *StCDPK7* was also found in the same phylogenetic group as *AtCDPK1*. Moreover, heterologous overexpression of *AtCPK1* in *Arabidopsis thaliana* was reported to upregulate disease resistance genes responsible for broad-spectrum protection against pathogen infection^47^. Here, the finding of *FaCDPK5* in the same phylogenetic group as *At*CDPK1 suggests that *Fa*CDPK5 may be involved in response to pathogen infection in strawberry. Further investigation is necessary to determine whether homologs possess similar functions.

Structural divergence, i.e. the presence and position of domains, as well as the organization of exons/introns can indicate evolutionary history within a gene family, and are also closely related to protein function^27,28^. As in barley and poplar^48,49^, the number of introns varied between one and seven in strawberry as well, indicating similarities in *CPDK* gene structure between different species.. Genes with similar intron phases share a common ancestor. A change in the intron phase reflects a divergence of homology between genes during evolution. The insertion or deletion of a small DNA fragment can alter the transcript, and thus lead to a change in gene function^50^. In Arabidopsis, the diversity of RNA structures was shown to arise due to a high number of deletions and additions of introns from the ancestor^28^. According to Mitall and co-workers^51^, variation in intron frequency in maize, rice, and sorghum may be one of the reasons for the functional diversity of CDPKs, as this variation increases the possibility of alternative splicing (AS) and exon shuffling. However, literature information on AS or RNA editing of the *CDPK* transcripts is very limited. Nishiyama and co-workers^52^ reported an AS of a *CDPK* gene, where EF-hand motif sequences were modified. Almadanin and co-workers^53^ showed that *OsCPK13*, *OsCPK17*, *OsCPK18,* and *OsCPK19* produced AS transcripts in rice, which encode truncated proteins lacking whole or partial domains with different biological functions. Finally, Ding and co-workers^54^ detected two transcripts of *CsCDPK1* from *C. sinensis* using RT-qPCR. The identification of AS variants in *CDPK* transcripts is particularly important for the development of genetically modified crops with improved biotic and abiotic stress tolerance. Therefore, future studies to identify splicing variants from CDPK genes, and the impact on their functional diversity are of relevance in this regard.

The specificity and gene expression patterns are clearly altered during plant development stages^55^. Here, *FaCDPK1* was not detected in young fruits. This result is in agreement with those reported by Llop-Tous^24^, who observed that *FaCDPK1* was only expressed at the final stage of fruit development (28 DAA). However, transcript levels increased as fruits whitened and during the maturation stage. Here, such increased expression during fruit ripening was observed only for *FaCDPK3*. Li L. et al*.*^45^ also observed similar results in cotton (*Gossypium raimondii*), in which only *GrCDPK11* expression increased during fruit development. Expression levels of the other *GrCDPKs* remained unchanged or decreased. Strawberry ripening process involves a series of molecular and physiological events leading to dramatic modifications in fruit size, color, texture, flavor, and aroma. Therefore, understanding the mechanisms underlying this process is essential to achieve optimal fruit quality as well as long shelf life. Transcriptomic, metabolomic, and gene silencing studies have shown that this process in non-climacteric fruits such as strawberry is ABA-dependent^56^, and triggered by a signaling cascade involving calcium (Ca^2+^) and calcium-dependent protein kinases (CDPKs)^15^.Increased expression of *FaCDPK3* here indicates a possible role during strawberry maturation. However, further studies are necessary to address ABA-dependence of this process.

In addition to the stage of development, different stimuli also determine which isoforms are activated or inactivated and their function. For example, *OsCDPK4* confers salt and drought tolerance to rice^11^, whereas *ZmCDPK1* plays a vital regulatory role in abiotic stress response of maize^38^. Drought and soil salinity negatively impact plant growth and crop production. Therefore, a better understanding of molecular response against these stresses is required to develop more resistant cultivars. Strawberry is generally considered moderately-sensitive to high salt levels. However, the cv. Camarosa used in this study is considered tolerant to drought and salt stress compared to other cultivars^57,58^. For example, the stress levels used in this study did not result in necrosis, extensive dehydration or death of plants (Figure S2). On the other hand, plants showed response to the inflicted stress as evidenced by RT-qPCR analysis results showing increases in expression levels of *FaCDPK1*, *FaCDPK3*, *FaCDPK4*, *FaCDPK5*, *FaCDPK6*, *FaCDPK8,* and *FaCDPK9* upon salt treatment, whereas those of *FaCDPK4*, *FaCDPK10* and *FaCDPK11* increased expression levels under drought conditions. This result suggests that *Fa*CDPK proteins play a role in the tolerance of this cultivar to salinity and drought stress, and corroborates the relationships between sequence, structure and protein function. *FaCDPK4*, *FaCDPK10* and *FaCDPK11* were grouped in the same phylogenetic subgroup (A) as *At*CDPK23, which was reported to be involved in drought tolerance of Arabidopsis^44^. Although osmotic stress is caused by both salt and drought stresses, excessive accumulation of Na^+^ and Cl^−^ ions may also occur under high salinity due to reduced water availability in the soil. Na^+^ and Cl^−^ accumulation is also detrimental to biochemical processes, and leads to chlorosis and necrosis. *FaCDPK4* was the only *CDPK* gene overexpressed under both experimental conditions. *FaCDPK4* may thus be associated with physiological responses triggered by both stresses, such as regulation of stomatal movement and repression of cell growth and photosynthesis. As an example, *AtCPK1* mediates salt and drought stress through decreased production of H_2_O_2_ and malondialdehyde, and increased accumulation of proline^59^. Since most of the CDPK genes were differentially-expressed between these stress conditions, and due to the varying substrate specificities of CDPK isoforms^46^, this result suggests that strawberry plants are able to respond differentially to these stresses, and the kinase cascade formed by CDPK proteins may have a role in this differential response.

Although plants use closely-related response mechanisms against salinity and drought, the effects these stresses on plants are not identical. Changes in the osmotic potential observed in both stresses seems to trigger the induction of different CDPKs. For example, tolerance to drought and salt stresses in *Morus atropurpurea* were reported to be related to the interaction of *MaCDPK1* with ABA response elements (MBS elements, ABRE and GARE-motif)^60^. However, the *CDPK* copies and the induction of phosphorylation cascade may differ between these two stresses. In *Poncirus trifoliata,* overexpression of *Ptr*CDPK10 resulted in enhanced drought stress tolerance compared to the wild type due to the interaction of *Ptr*CDPK10 with ascorbate peroxidase (*Ptr*APX), which led to a reduction in ROS accumulation^61^. In contrast, *OsCDPK21* was shown to play a crucial role in adaptation to salt stress in rice through phosphorylation of *Os*GF14. In grapevine, *VaCPK21* may act as a positive regulator involved in the response to salt stress^62^. *Ma*CDPK3^63^and *AtCDPK27* perform similar functions in banana and Arabidopsis, respectively^64^.

Plant responses to stress conditions are known to occur through various signal transduction networks, and abscisic acid (ABA) plays a crucial role in these responses. ABA mainly functions as a regulator of plant water balance and osmotic stress tolerance^65^. CDPK isoforms were also shown to differ in their Ca^2+^ affinity, and calcium influx generated by ABA is one of the determinants of the induction of CDPKs^15^. As an example, Zhang H. et al*.*^22^ performed an analysis of the transcripts of 21 *BnaCDPK* genes in canola seedlings following different treatments under abiotic stress conditions, and found that different *CDPK* genes may participate in the signaling processes of a single stress factor, and a single *CDPK* gene likely plays a role in multiple stress responses. In rice, *OsCDPK4* was reported to play a positive role in tolerance to saline and water stresses through membrane protection against lipid peroxidation^11^; where as *Os*CPK12 was found to increase salt tolerance by reducing ROS levels, suggesting that different copies work on multiple signaling pathways to positively regulate salt tolerance^66^.

Here, *FaCDPK4* and *FaCDPK11*presented the highest levels of expression against water stress, and these genes were also the copies with best response to ABA treatment. This suggests that these genes play important roles in water stress response, and the response mechanism is ABA-dependent. This relationship was also observed in Arabidopsis, in which mutants lacking *AtCDPK10* expression were impaired in their ability to inhibit ABA-induced stomatal opening. In a study by Zhu et al*.*^67^, AtCPK4 and AtCPK11 were shown to positively regulate ABA signaling through the phosphorylation of stress-responsive transcription factors *ABF1* and *ABF4*. ABA was also shown to activate calcium-dependent protein kinase (*ACPK1*) in the grape mesocarp by means of a complex mechanism involving the influx of calcium to the cytosol^68^. Therefore, the variations in ABA-induced Ca^2+^ signal discharge (concentration, size, temporal distribution of spikes or waves) may result in specific signal transduction to induce the expression of specific *CDPK* genes. Consequently, the induced CDPK protein would play specific roles depending on the inflicted stress type through phosphorylation of specific substrates^46^.

**Figure 8:**
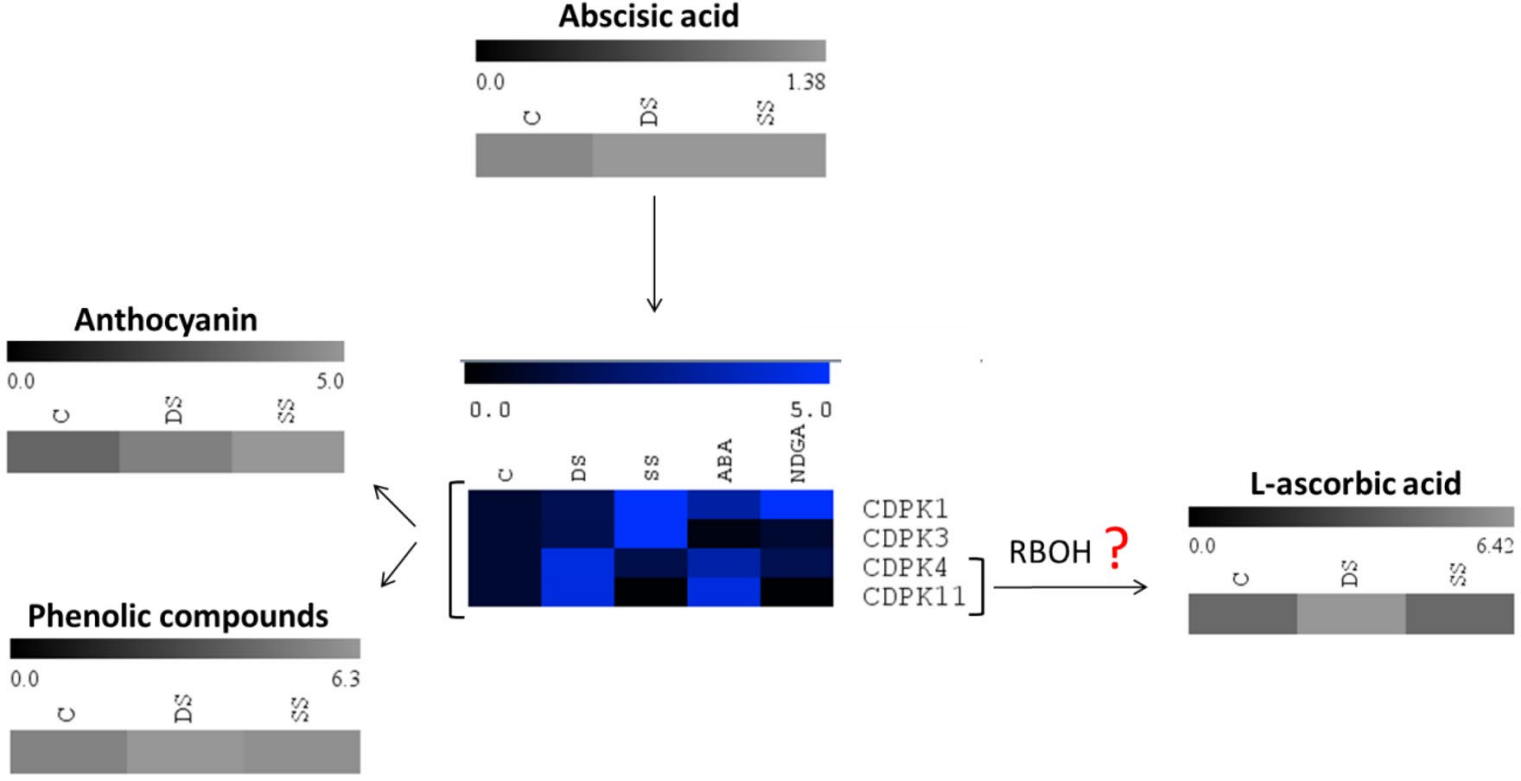
Simplified scheme of possible CDPK actuations under osmotic stress conditions.

We previously observed that salt and drought stress increase the content of phenolic and anthocyanin compounds as well as the expression of genes in related metabolic pathways^25,69^. ABA content was also observed to increase in strawberry fruits under saline and drought stresses; however, ABA biosynthesis related genes (*FaNCEDs*, *FaCYP707As*, *FaGTs,* and *FaBGs*) were mostly upregulated only in drought stressed fruits^25^. Similarly, only the drought stress increased the concentrations of ascorbic acid, sugars, and methylsyringin, whereas salt stress induced the synthesis of amino acids in the fruits^26,70^. A mechanism of salt and drought response in strawberry fruits based on these previous results and the gene expression data from this study is shown in Fig. 8. Saline stress response was found to be mediated by several *FaCDPKs*, in particular *FaCDPK1* and *FaCDPK3* through an ABA-independent mechanism. In contrast, *FaCDPK4* and *FaCDPK11* were found to be involved in drought stress tolerance, and utilize ABA as the signaling molecule to increase the content of phenolics, anthocyanins, ascorbic acid, and sugar compounds. The crosstalk between CDPKs, ABA, phenylpropanoids and sugar was recently reviewed by Vighi et al.^15^, and the influence of ABA in the ascorbic acid metabolism of strawberry fruits was discussed by Li et al*.*^71^.

The results above suggest an involvement of *FaCDPKs* in the response to salt and drought stresses. Several other studies have reported that CDPKs play important roles in tolerance to abiotic stress^72,73^. However, the underlying biochemical mechanisms are still unknown. Determining protein–protein interactions is an essential step towards understanding the mechanisms involved in the development of tolerance to abiotic stresses. Stress response involves the production of reactive oxygen species (ROS), and may induce the flow of calcium into the cell. Both signaling molecules induce downstream signaling events that result in the activation of Ca^2+^ sensor proteins including CDPK as well as the ROS-producing enzyme andr espiratory oxidase homolog proteins (RBOH)^15^.

*FaCDPK4* and *FaCDPK11* genes were likely involved in response to abiotic stress, especially drought, and the interaction network based on the co-expression analysis revealed that *FaCDPK4* and *FaCDPK11*were possibly associated with RBOHs. RBOHs are integral membrane proteins that generate superoxide anions, which are then converted to H_2_O_2._ Moreover, RBOH proteins contain two calcium-binding EF hand motifs, and multiple phosphorylation sites at their N-termini that participate in regulation of enzyme activity. In addition to their roles in responses to abiotic stresses, RBOH are play fundamental roles in several metabolic processes such as cellular growth and hypersensitivity responses^74^. RBOHs are differentially expressed according to the tissue and developmental stage^75^. Moreover, different stimuli determine which isoforms will be activated/inactivated^7^. For example, in Arabidopsis, *AtRBOHD* was found to be responsible for the generation of ROS in response to pathogen infection^77^. In strawberry, *FvRBOHA* and *FvRBOHD* are required for accumulation of ROS during plant defense response against cold stress^78^. Similarly, the expression levels of *VvRBOHA*, *VvRBOHB,* and *VvRBOHC1* were significantly increased in grapes upon salt and drought stress treatments. In addition, *VvRBOHB* was strongly upregulated by exogenous ABA treatments^79^. Here, *FaCDPK4* and *FaCDPK11*were found to be responsive to drought and ABA treatments as well, as shown in Fig. 8.This suggests that stress response involves the cellular uptake/synthesis of ABA and Ca^2+^, and both of these signaling molecules may induce downstream signaling events that result in increased production of H_2_O_2_ by RBOH, and the consequent induction of ascorbic acid to mitigate the production of ROS^80^.

Besides the RBOH proteins, a calcineurin B-like protein (CBL) was highlighted in the interaction network of *Fa*CDPK4. CBL are calcium sensor proteins that integrate and interact specifically with a family of protein kinases (CIPKs)^81^. CBL-CIPKs are involved in immune and abiotic stress responses in addition to plant growth and development^33,82,83^. CBL-CIPKs mediated stress response was also shown to be ABA-dependent in several studies^84,85^.

*Fa*CDPK4 also showed a similar expression pattern to that of thiamine pyrophosphokinase (TPK), a protein responsible for thiamine pyrophosphate activation, and an essential cofactor of numerous enzymes participating in the metabolism of carbohydrates and amino acids^86,87^. Moreover, thiamine also confers systemic acquired resistance (SAR) to several plants such as rice, tobacco, tomato (*Lycopersicon esculentum*), cucumber (*Cucumis sativus*), and Arabidopsis^88^. In Arabidopsis, it was demonstrated that this process is dependent on salicylic acid and Ca^2+^ related signaling pathways^89^.

Another protein identified in the *Fa*CDPK4 interaction network is the guard cell S-type anion channel (SLAC). SLAC plays important roles in stress and hormone signaling as well as plant growth and development^90^. SLCA1 is also an essential protein for stomatal aperture, and activated by ABA and cytosolic Ca^2+^. SLAC1 channels are activated by CDPKs as well^91^. As an example, *A. thaliana* guard cells with silenced*CDPK3* and *CDPK6*genes showed reduced activation of the ion channels of Ca^2+^ and ABA, and stomatal closing was partially impaired^92^.

Similar to that of *FaCDPK4*, the most prominent proteins in the protein interaction network of *FaCDPK11*were RBOHs. However, several other proteins were also highlighted, such as the serine decarboxylase (SDC). SDC is responsible for conversion of serine to ethanolamine, a phospholipid precursor in plants^93^. Phospholipids are essential components of biological membranes and signal transduction cascades in plants^94^. Phospholipids also influence the sensitivity of CDPK to calcium. Interaction of kinase with phospholipids was shown to lower its requirement for calcium in maize^95^. Moreover, *FaCDPK11*interacted with the probable transcription factor (POSF)/basic leucine zipper (bZIP). bZIPs are involved in various cellular processes related to plant development, environmental signaling and stress response^96^. bZIP is part of a complex signaling network involving ABA and CDPK in abiotic stress response. In the presence of ABA, the PYR class of receptors sequesters PP2C, which in turn releases SnRK_2_. As a result, SnRK_2_ activates CDPK, and results in a phosphorylation cascade involving bZIP^97^. In Arabidopsis, three CDPKs (CDPK4, CDPK6, and CDPK33) efficiently phosphorylate bZIP^98^.

*Fa*CDPK11 may also be associated with the LRR receptor-like serine/threonine-protein kinase (GSO). Receptor-like kinases are transmembrane proteins involved in multiple physiological processes, including development, stress responses, hormone perception, and disease resistance^99^. Under abiotic stress conditions, GSO perceives the extracellular ligands, and activates downstream pathways via phosphorylation of intracellular serine/threonine kinase domains^100^. GSO also plays important roles in the ripening process together with ABA. *FaRIPK1* silencing inhibited ripening and reduced the expression of several genes involved in softening, sugar production, pigmentation, and ABA biosynthesis and signaling in strawberry plants^101^.

In summary, all proteins highlighted in the *FaCDPK4* and *FaCDPK11* interaction networks have well-documented roles in responses of plants to stress via Ca^2+^ signaling. These responses directly or indirectly involve the phytohormone ABA. The only protein in the *FaCDPK11* interaction network with no evident crosstalk with ABA and stress response is the tubulin-folding cofactor (TFCA). TFCA is involved in microtubule biogenesis, and thereby directly influences cell growth^102^. In Arabidopsis, TFCA requirement was demonstrated for the formation of trichome cells^103^. However, additional in vivo studies are necessary to validate the existence of this interaction predicted by in silico interaction network analysis.

In this study, nine new copies of *CDPKs* were identified in *F. ananassa* genome via genome structure analysis and phylogenetic characterization*.* The expression profiles of the identified *FaCDPKs* during fruit development and ripening indicated that only *FaCDPK3* expression increased during fruit ripening. *FaCDPK* genes were also found to be involved in the response to osmotic stresses: *FaCDPK1* and *FaCDPK3* in response against salt, and *FaCDPK4* and *FaCDPK11* against drought. The expression levels of the latter two genes were also significantly influenced by ABA treatment, suggesting the involvement of this phytohormone in the drought response mediated by these proteins. Moreover, the protein–protein interaction network analysis highlighted proteins involved in the ABA-dependent response to plant stress via Ca^2+^ signaling (RBOHs in particular). Future functional genomic studies will allow elucidating the role of *Fa*CDPKs in plants in order to develop biofortified fruits and stress-tolerant plants. Since responses of CDPK isoforms varied according to the inflicted stress type, further studies on the effect of distinct waves or signal signatures of calcium under the presence of ABA or an ABA inhibitor. Moreover, identification of phosphorylation targets of each CDPK may help identify the mechanism involved in induction of individual CDPK isoforms under a wide spectrum of conditions.

## Methods

### Identification of CDPK genes in strawberry

To search for the putative *FaCDPK* genes, the SRA database under the accession number SRP148865 was used. The search for homologous sequences in these datasets was performed using non-redundant sequences from National Center for Biotechnology Information (NCBI) (https://www.ncbi.nlm.nih.gov/), and from the Swiss-prot database (http: // www. expasy.ch/sprot/) by applying the BLASTX program (e-value < 1e^−6^). Putative sequences were translated into peptides from the largest open reading frame using the ORF Finder tool (https://www.ncbi.nlm.nih.gov/gorf/gorf.html). To verify the reliability of these candidate sequences, the typical domains of CDPKs, including the Ca^2+^ binding domain, the N-terminal and the autoinhibitory domain, were searched using the PROSITE tool (https://prosite.expasy.org/). The N-myristoylation motif and the palmitoylation site were predicted by Myristoylator (https://web.expasy.org/myristoylator/)^104^ and CSS-Plam program^105^, respectively. Subcellular localization prediction was performed in Plant-PLoc (https://www.csbio.sjtu.edu.cn/bioinf/plant/), ChloroP v 1.1 (https://www.cbs.dtu.dk/services/ChloroP/), TMHMM v. 2.0 (https://www.cbs.dtu.dk/services/TMHMM/), and DeepLoc v. 1.0 (https://www.cbs.dtu.dk/services/DeepLoc/).

### Gene structure and phylogenetic analysis

The genome of *F. ananassa* (scaffolds) was downloaded and used along with the cDNAs of each of the genes to evaluate the pattern of exons and introns distribution. This distribution was performed using the GSDS bioinformatics tool (https://gsds.cbi.pku.edu.cn/). To search for conserved motifs within the *Fa*CDPK proteins, the online MEME tool (https://meme.nbcr.net/meme/cgi-bin/meme.cgi) was used to find similar sequences shared by these members.

For construction of the phylogenetic tree, the putative *Fa*CDPK sequences along with CDPK sequences previously described in *A. thaliana* (*At*CDPKs) were aligned in ClustalW 1.8.1 using the standard parameters. This alignment was used to infer the phylogenetic relationships of the CDPK genes. The phylogenetic tree was constructed in the MEGA6 program, using the Neighbor Joining grouping method and different model substitutions: *p*-distance, Jones-Taylor-Thornton (JTT) model, Dayhoff model, Equalimput model and Poisson model. All analyzes were performed with bootstrap values of 1000^106^.

### Synteny analysis and chromosome localization

*FaCDPK* genes were mapped to strawberry chromosomes based on information of *Fragaria vesca* available at the National Center for Biotechnology Information (NCBI), obtained by using BLASTP with the reference E < 1e^-5^. The map was drafted using Mapchart software (https://www.wageningenur.nl/en.htm).The syntenic blocks were used for constructing a synteny analysis map within the strawberry (*Fragaria vesca*) genome and Arabidopsis. Diagrams were generated using the Circos program (version 0.69) (https://circos.ca/). Based on the phylogenetic tree analysis results, the synonymous (Ks) and non-synonymous (Ka) nucleotide substitutions between orthologous and paralogous gene pairs were calculated using ClustalW and PAL2NAL.

### Plant materials and stress treatments

The study was conducted on a greenhouse, using the cultivar Camarosa, as it is extensive used in south Brazil, and because of its good agronomic performance. The seedlings were planted and grown in 9 L pots, containing soil (Ultisoil) and vermiculite (in a proportion of 3:1). The fertilization and irrigation were performed according to Galli, V. et al*.*^107^. The relative humidity in each pot was monitored using a hygrometer order to maintain between 16 and 19%, without water leaching.

The experiment was composed by five treatments with six replicates per treatment and ten plants per replicate. The treatments were as follows: control/normal irrigation (C), corresponding to 100% crop evapotranspiration (ETc); drought stress (DS), corresponding to 85% crop ETc; the salt stress (SS), with 80 mM NaCl; 200 µM of abscisic acid (ABA); and 50 µM of nordihydroguaiaretic acid (NDGA). ABA and NDGA treatments were imposed by spraying the foliage of seedlings. An approximate pool of ten ripe fruits (fully red, according to Jia, H. et al.^56^) by replicate were harvested.

Fruits from six developmental stages were also sampled (15 uniformly sized fruits at each stage) from plants cultivated under the same conditions as the control treatment. The stages corresponded to 7 (small green, SG), 14 (big green, BG), 18 (degreening DG), 21 (white, W), 24 (partial red, PR), and 28 (full red, FR) days after anthesis (DAA), as previously reported Jia, H .et al*.*^56^.

All samples were immediately frozen in liquid nitrogen and stored at -80 °C until further analysis.

### Gene relative expression analysis

Total RNA isolation, cDNA synthesis and RT-qPCR amplification were performed as described by Galli, V. et al*.*^107^. The reference genes PIRUV_DESCARB (pyruvate decarboxylase), DBP (DNA binding protein), HISTH4 (histone H4) were used to normalize transcription levels, as proposed by the authors. The primers used are presented in Table 2. The relative expression data were calculated according to the 2^-ΔΔCq^ method. The analysis was performed for four biological replicates and three analytical replicates. The Heat map representation was used to estimate the fold-change of gene expression using the software Mev 4.8.1 (https://sourceforge.net/projects/mev-tm4/files/mev-tm4/). The results were submitted to analysis of variance (ANOVA) and when significant (p ≤ 0.05) were submitted to a comparison of means by Tukey test at 5% of probability. Statistical analyzes were performed using SAS software.

**Table 2:** Sequence of primers generated from the selected sequences of strawberry (*Fragaria x ananassa*) CDPK.

**Gene name**	**Primer forward**	**Primer reverse**
FaCDPK1	GGTGAAACAGTGAGAGCAAGGC	AAATCCCTCTTGTATGCCAGC
FaCDPK2	TGGATTTCACCGAGTTTGTTG	AGGCTAATCTTCCCGTCTTTGT
FaCDPK3	CACCAAACCCAAACTCAACATT	TATGGCGGTCAGAGTAGTAGGAGT
FaCDPK4	TCGGGTTGTCAGTGTTCATCG	CTCAAAGTCAATCTCTCCCTCCAA
FaCDPK5	GCAGGCAGCAGATGTAGATAATAGC	AACATCCTCTACACCAAACTCCTCA
FaCDPK6	TGACAAGGATGGAAGTGGCTAT	CGCCCATCCTTGTCAGTGT
FaCDPK7	AAGCAACTGAGAGCAATGAATAAGC	TTGCCTGACTTCCGTTTCTGAT
FaCDPK8	CTGCTAAGGATGAGAGTTCGCC	ACACTCCATACATCAGCCTCCG
FaCDPK9	CGACAAAGATGGAAGCGGG	CCATTTCCCTTCCTCATCATTG
FaCDPK10	TGATGTGGATGGAAATGGAACC	TAATCGTTTGTTCGTCACCCAT
FaCDPK11	TCCAACACAACAACCTCAGAAAGT	CTTCCGAGTTCCTTGCCCA

### Network interaction

Specific interaction network using textmining, databases, co-expression, neighborhood, gene fusion, co-occurrence and experimental evidences, of *FaCDPK4* and *FaCDPK11* was constructed using online STRING 10 (https://string-db.org/).

## Supplementary information

Supplementary information

Supplementary information

## References

[1] Krasensky J, Jonak C (2012). Drought, salt, and temperature stress-induced metabolic rearrangements and regulatory networks. J. Exp. Bot..

[2] Hasanuzzaman M, Nahar K, Alam MM, Roychowdhury R, Fujita M (2013). Physiological, biochemical, and molecular mechanisms of heat stress tolerance in plants. Int. J. Mol. Sci..

[3] Ray SD (2017). Ca 2 +, The miracle molecule in plant hormone signaling during abiotic stress. Mech. Plant Horm. Signal. Stress.

[4] Edel KH, Marchadier E, Brownlee C, Kudla J, Hetherington AM (2017). The evolution of calcium-based signalling in plants. Curr. Biol..

[5] Nomura H, Shiina T (2014). Calcium signaling in plant endosymbiotic organelles: Mechanism and role in physiology. Mol. Plant.

[6] Liese A, Romeis T (2013). Biochemical regulation of in vivo function of plant calcium-dependent protein kinases (CDPK). Biochim. Biophys. Acta Mol. Cell Res..

[7] Valmonte GR, Arthur K, Higgins CM, Macdiarmid RM (2014). Calcium-dependent protein kinases in plants: Evolution, expression and function. Plant Cell Physiol..

[8] Gao X, Cox K, He P (2014). Functions of calcium-dependent protein kinases in plant innate immunity. Plants.

[9] Boudsocq M, Sheen J (2013). CDPKs in immune and stress signaling. Trends Plant Sci..

[10] Hamel LP, Sheen J, Séguin A (2014). Ancient signals: Comparative genomics of green plant CDPKs. Trends Plant Sci..

[11] Campo S (2014). Overexpression of a calcium-dependent protein kinase confers salt and drought tolerance in rice by preventing membrane lipid peroxidation. Plant Physiol..

[12] Bundó M, Coca M (2016). Enhancing blast disease resistance by overexpression of the calcium-dependent protein kinase OsCPK4 in rice. Plant Biotechnol. J..

[13] Wei S (2014). A rice calcium-dependent protein kinase OsCPK9 positively regulates drought stress tolerance and spikelet fertility. BMC Plant Biol..

[14] Zou JJ (2015). Arabidopsis calcium-dependent protein kinase8 and CATALASE3 function in abscisic acid-mediated signaling and H<inf>2</inf>O<inf>2</inf> homeostasis in stomatal guard cells under drought stress. Plant Cell.

[15] Vighi IL, Crizel RL, Perin EC, Rombaldi CV, Galli V (2019). Crosstalk during fruit ripening and stress response among abscisic acid, calcium-dependent protein kinase and phenylpropanoid. CRC. Crit. Rev. Plant Sci..

[16] Aleynova-Shumakova OA, Dubrovina AS, Manyakhin AY, Karetin YA, Kiselev KV (2014). VaCPK20 gene overexpression significantly increased resveratrol content and expression of stilbene synthase genes in cell cultures of Vitis amurensis Rupr. Appl. Microbiol. Biotechnol..

[17] Allwood EG, Davies DR, Gerrish C, Ellis BE (1999). Phosphorylation of phenylalanine ammonia-lyase: Evidence for a novel protein kinase and identication of the phosphorylated residue. FEBS Lett..

[18] Asano T, Tanaka N, Yang G, Hayashi N, Komatsu S (2005). Genome-wide identification of the rice calcium-dependent protein kinase and its closely related kinase gene families: Comprehensive analysis of the CDPKs gene family in rice. Plant Cell Physiol..

[19] Hrabak EM (2003). The arabidopsis CDPK-SnRK superfamily of protein kinases. Plant Physiol..

[20] Cheng S, Willmann MR, Chen H, Sheen J (2002). Update on calcium signaling through protein kinases. The arabidopsis calcium-dependent protein kinase gene family 1. Plant Physiol..

[21] Wang JP, Xu YP, Munyampundu JP, Liu TY, Cai XZ (2016). Calcium-dependent protein kinase (CDPK) and cdpk-related kinase (CRK) gene families in tomato: Genome-wide identification and functional analyses in disease resistance. Mol. Genet. Genom..

[22] Zhang H (2014). Identification and characterization of CBL and CIPK gene families in canola (Brassica napus L). BMC Plant Biol..

[23] Jia HF (2013). Type 2C protein phosphatase ABI1 is a negative regulator of strawberry fruit ripening. J. Exp. Bot..

[24] Llop-Tous I (2002). Characterization of a strawberry cDNA clone homologous to calcium-dependent protein kinases that is expressed during fruit ripening and affected by low temperature. J. Exp. Bot..

[25] Perin EC (2019). ABA-dependent salt and drought stress improve strawberry fruit quality. Food Chem..

[26] Galli V (2018). Transcriptome analysis of strawberry (Fragaria × ananassa) fruits under osmotic stresses and identification of genes related to ascorbic acid pathway. Physiol. Plant..

[27] Kudla J, Batistic O, Hashimoto K (2010). Calcium signals: the lead currency of plant information processing. Plant Cell.

[28] Boudet N, Aubourg S, Toffano-nioche C, Kreis M, Lecharny A (2001). Evolution of intron/exon structure of DEAD helicase family genes evolution of intron/exon structure of DEAD helicase family genes in arabidopsis, caenorhabditis, and drosophila. Genome Res..

[29] Juretic N, Hoen DR, Huynh ML, Harrison PM, Bureau TE (2005). The evolutionary fate of MULE-mediated duplications of host gene fragments in rice. Genome Res..

[30] Li J (2009). Correlation between Ka/Ks and Ks is related to substitution model and evolutionary lineage. J. Mol. Evol..

[31] Zhao F (2018). Identification of basic/helix-loop-helix transcription factors reveals candidate genes involved in anthocyanin biosynthesis from the strawberry white-flesh mutant. Sci. Rep..

[32] Hetherington A, Trewavas A (1982). Calcium-dependent protein kinase in pea shoot membranes. FEBS Lett..

[33] Mohanta T (2019). Molecular players of EF-hand containing calcium signaling event in plants. Int. J. Mol. Sci..

[34] Zhao Y (1994). Calcium-binding properties of a calcium-dependent protein kinase from Plasmodium falciparum and the significance of individual calcium-binding sites for kinase activation. Biochemistry.

[35] Martín ML, Busconi L (2000). Membrane localization of a rice calcium-dependent protein kinase (CDPK) is mediated by myristoylation and palmitoylation. Plant J..

[36] Lu SX, Hrabak EM (2013). The myristoylated amino-terminus of an Arabidopsis calcium-dependent protein kinase mediates plasma membrane localization. Plant Mol. Biol..

[37] Li AL (2008). Evolutionary and functional study of the CDPK gene family in wheat (*Triticum aestivum* L.). Plant Mol. Biol..

[38] Wang CT, Shao JM (2013). Characterization of the ZmCK1 gene encoding a calcium-dependent protein kinase responsive to multiple abiotic stresses in maize. Plant Mol. Biol. Rep..

[39] Asai S (2013). The variable domain of a plant calcium-dependent protein kinase (CDPK) confers subcellular localization and substrate recognition for NADPH oxidase. J. Biol. Chem..

[40] Hu W (2016). Genome-wide survey and expression analysis of the calcium-dependent protein kinase gene family in cassava. Mol. Genet. Genom..

[41] Zhang M (2020). Genome-wide investigation of calcium-dependent protein kinase gene family in pineapple: Evolution and expression profiles during development and stress. BMC Genom..

[42] Chen F (2013). The evolutionary history and diverse physiological roles of the grapevine calcium-dependent protein kinase gene family. PLoS ONE.

[43] Ray S, Agarwal P, Arora R, Kapoor S, Tyagi AK (2007). Expression analysis of calcium-dependent protein kinase gene family during reproductive development and abiotic stress conditions in rice (*Oryza sativa* L ssp indica). Mol. Genet. Genom..

[44] Ma SY, Wu WH (2007). AtCPK23 functions in Arabidopsis responses to drought and salt stresses. Plant Mol. Biol..

[45] Li LB (2015). Genome-wide analysis of the calcium-dependent protein kinase gene family in Gossypium raimondii. J. Integr. Agric..

[46] Fantino E, Segretin ME, Santin F, Mirkin FG, Ulloa RM (2017). Analysis of the potato calcium-dependent protein kinase family and characterization of StCDPK7, a member induced upon infection with Phytophthora infestans. Plant Cell Rep..

[47] Coca M, San Segundo B (2010). AtCPK1 calcium-dependent protein kinase mediates pathogen resistance in Arabidopsis. Plant J..

[48] Zuo R (2013). Genome-wide identification, classification, and expression analysis of CDPK and its closely related gene families in poplar (Populus trichocarpa). Mol. Biol. Rep..

[49] Fedorowicz-Strońska O, Koczyk G, Kaczmarek M, Krajewski P, Sadowski J (2017). Genome-wide identification, characterisation and expression profiles of calcium-dependent protein kinase genes in barley (*Hordeum vulgare* L.). J. Appl. Genet..

[50] Long M, de Souza SJ, Rosenberg C, Gilbert W (1998). Relationship between ‘proto-splice sites’ and intron phases: Evidence from dicodon analysis. Proc. Natl. Acad. Sci..

[51] Mittal S (2017). Comparative analysis of CDPK family in maize, Arabidopsis, rice, and sorghum revealed potential targets for drought tolerance improvement. Front. Chem..

[52] Nishiyama R (1999). Two mRNA species encoding calcium-dependent protein kinases are differentially expressed in sexual organs of Marchantia polymorpha through alternative splicing. Plant Cell Physiol..

[53] Almadanim MC (2018). The rice cold-responsive calcium-dependent protein kinase OsCPK17 is regulated by alternative splicing and post-translational modifications. Biochim. Biophys. Acta Mol. Cell Res..

[54] Ding Y (2020). Alternative splicing in tea plants was extensively triggered by drought, heat and their combined stresses. PeerJ..

[55] Simeunovic A, Mair A, Wurzinger B, Teige M (2016). Know where your clients are: Subcellular localization and targets of calcium-dependent protein kinases. J. Exp. Bot..

[56] Jia H-F (2011). Abscisic acid plays an important role in the regulation of strawberry fruit ripening. Plant Physiol..

[57] Al-Shorafa W, Mahadeen A, Al-Absi K (2014). Evaluation for salt stress tolerance in two strawberry cultivars. Am. J. Agric. Biol. Sci..

[58] Ghasemi H, Amiri Fahliani R, Kavoosi B, Dehdari M (2018). Response of some strawberry (Fragaria ananssa Duch) cultivars to deficit irrigation regarding leaf area and some quantitative and qualitative characteristics of fruit. J. Sci. Technol. Greenh. Cult..

[59] Huang K (2018). Arabidopsis calcium-dependent protein kinase AtCPK1 plays a positive role in salt/drought-stress response. Biochem. Biophys. Res. Commun..

[60] Cao Z (2020). Functional characteristics of a calcium-dependent protein kinase (MaCDPK1) enduring stress tolerance from Morus atropurpurea Roxb. Plant Cell. Tissue Organ Cult..

[61] Meng L, Zhang Q, Yang J, Xie G, Liu JH (2020). PtrCDPK10 of Poncirus trifoliata functions in dehydration and drought tolerance by reducing ROS accumulation via phosphorylating PtrAPX. Plant Sci..

[62] Dubrovina AS, Kiselev KV, Khristenko VS, Aleynova OA (2016). VaCPK21, a calcium-dependent protein kinase gene of wild grapevine Vitis amurensis Rupr, is involved in grape response to salt stress. Plant Cell. Tissue Organ Cult..

[63] Wang Z, Li J, Jia C, Xu B, Jin Z (2016). Molecular cloning and expression analysis of eight calcium-dependent protein kinase (CDPK) genes from banana (Musa acuminata L. AAA group, cv. Cavendish). South Afr. J. Bot..

[64] Zhao R (2015). The Arabidopsis Ca^2+^-dependent protein kinase CPK27 is required for plant response to salt-stress. Gene.

[65] Dar NA (2017). Abscisic acid: A key regulator of abiotic stress tolerance in plants. Plant Gene.

[66] Asano T (2012). A rice calcium-dependent protein kinase OsCPK12 oppositely modulates salt-stress tolerance and blast disease resistance. Plant J..

[67] Zhu S-Y (2007). Two calcium-dependent protein Kinases, CPK4 and CPK11, regulate abscisic acid signal transduction in *Arabidopsis*. Plant Cell.

[68] Yu X, Li M, Gao G, Feng H (2006). Abscisic acid stimulates a calcium-dependent protein kinase in grape berry. Plant Physiol..

[69] Galli V (2016). Mild salt stress improves strawberry fruit quality. LWT Food Sci. Technol..

[70] Antunes ACN (2020). Untargeted metabolomics of strawberry (Fragaria x ananassa ‘Camarosa’) fruit from plants grown under osmotic stress conditions Ana. J. Sci. Food Agric..

[71] Li D (2015). Comparative transcriptome analysis reveals the influence of abscisic acid on the metabolism of pigments, ascorbic acid and folic acid during strawberry fruit ripening. PLoS ONE.

[72] Asano T, Hayashi N, Kikuchi S, Ohsugi R (2012). CDPK-mediated abiotic stress signaling. Plant Signal. Behav..

[73] Kardile HB (2018). Calcium-dependent protein kinases (CDPK) in abiotic stress tolerance. J. Plant Sci. Res..

[74] Foreman J (2003). Reactive oxygen species produced by NADPH oxidase regulate plant cell growth. Nature.

[75] Torres MA, Dangl JL (2005). Functions of the respiratory burst oxidase in biotic interactions, abiotic stress and development. Curr. Opin. Plant Biol..

[76] Chapman JM, Muhlemann JK, Gayomba SR, Muday GK (2019). RBOH-dependent ROS synthesis and ROS scavenging by plant specialized metabolites to modulate plant development and stress responses. Chem. Res. Toxicol..

[77] Angel M, Dangl JL, Jones JDG, Torres MA (2015). Arabidopsis gp91phox homolc AtrbohF are required for acc umulation of gues AtrbohD and reactive oxygen intermediat !s in the plant defense response. Proc. Natl. Acad. Sci..

[78] Zhang Y (2018). Identification of NADPH oxidase family members associated with cold stress in strawberry. FEBS Open Bio.

[79] Cheng C (2013). Genome-wide analysis of respiratory burst oxidase homologs in grape (Vitis vinifera L.). Int. J. Mol. Sci..

[80] Choudhury FK, Rivero RM, Blumwald E, Mittler R (2017). Reactive oxygen species, abiotic stress and stress combination. Plant J..

[81] Batistič O, Kudla J (2009). Plant calcineurin B-like proteins and their interacting protein kinases. Biochim. Biophys. Acta Mol. Cell Res..

[82] Luo Q (2017). BdCIPK31, a Calcineurin B-like protein-Interacting protein kinase, regulates plant response to drought and salt stress. Front. Plant Sci..

[83] Chen L (2013). Arabidopsis CBL-interacting protein kinase (CIPK6) is involved in plant response to salt/osmotic stress and ABA. Mol. Biol. Rep..

[84] Pandey GK (2015). Calcineurin B-like protein-interacting protein kinase CIPK21 regulates osmotic and salt stress responses in Arabidopsis. Plant Physiol..

[85] Tripathi V, Parasuraman B, Laxmi A, Chattopadhyay D (2009). CIPK6, a CBL-interacting protein kinase is required for development and salt tolerance in plants. Plant J..

[86] Roje S (2007). Vitamin B biosynthesis in plants. Phytochemistry.

[87] Rapala-kozik M, Go A, Kujda M (2009). Plant Physiology and Biochemistry Enzymes that control the thiamine diphosphate pool in plant tissues Properties of thiamine pyrophosphokinase and thiamine- (di) phosphate phosphatase purified from Zea mays seedlings. Plant Physiol. Biochem. J..

[88] Ahn I, Kim S, Lee Y (2005). Vitamin B 1 functions as an activator of plant disease resistance 1. Plant Physiol..

[89] Ahn I, Kim S, Lee Y, Suh S (2007). Vitamin B 1-induced priming is dependent on hydrogen peroxide and the NPR1 gene in arabidopsis 1. Plant Physiol..

[90] Chen G (2018). Genomics Genome-wide survey and expression analysis of the SLAC/SLAH gene family in pear (Pyrus bretschneideri) and other members of the Rosaceae. Genomics.

[91] Assmann SM, Jegla T (2016). Guard cell sensory systems: recent insights on stomatal responses to light, abscisic acid, and CO 2. Curr. Opin. Plant Biol..

[92] Mori IC (2006). CDPKs CPK6 and CPK3 function in ABA regulation of guard cell S-type anion- and Ca 2 þ-permeable channels and stomatal closure. PLoS Biol..

[93] Liu Y (2018). Arabidopsis serine decarboxylase 1 (SDC1) in phospholipid and amino acid metabolism plant materials and growth conditions. Front. Plant Sci..

[94] Nakamura Y (2017). Plant phospholipid diversity: emerging functions in metabolism and protein–lipid interactions. Trends Plant Sci..

[95] Szczegielniak J, Liwosz A, Jurkowski I, Loog M, Harmon AC (2000). Calcium-dependent protein kinase from maize seedlings activated by phospholipids. Fed. Eur. Biochem. Soc..

[96] Droge-Laser W, Snoek BL, Snel B, Weiste C (2018). The Arabidopsis bZIP transcription factor family—an update. Curr. Opin. Plant Biol..

[97] Banerjee A, Roychoudhury A (2017). Abscisic-acid-dependent basic leucine zipper ( bZIP ) transcription factors in plant abiotic stress. Protoplasma.

[98] Kawamoto N, Sasabe M, Endo M, Machida Y, Araki T (2015). Responsible for the phosphorylation of a florigen complex formation. Sci. Rep..

[99] Passricha N, Saifi SK, Singh R, Kharb P, Tuteja N (2019). Receptor-like kinases control the development, stress response, and senescence in plants. Senescence Signal. Control Plants.

[100] Feng L, Gao Z, Xiao G, Number FO (2014). Leucine-rich repeat receptor-like kinase FON1 regulates drought stress and seed germination by activating the expression of ABA-responsive genes in Rice. Plant Mol. Biol. Report..

[101] Hou B, Xu C, Shen Y (2018). RESEARCH PAPER A leu-rich repeat receptor-like protein kinase, FaRIPK1, interacts with the ABA receptor. FaABAR.

[102] Kirik V (2002). Functional analysis of the tubulin-folding cofactor C in *Arabidopsis thaliana*. Curr. Biol..

[103] Chen L (2016). Interacts with KCBP / ZWICHEL to regulate trichome cell shape in Arabidopsis thaliana. PLoS Genet..

[104] Bologna G, Yvon C, Duvaud S, Veuthey AL (2004). N-terminal myristoylation predictions by ensembles of neural networks. Proteomics.

[105] Ren J (2008). CSS-Palm 20: An updated software for palmitoylation sites prediction. Protein Eng. Des. Sel..

[106] Tamura K, Dudley J, Nei M, Kumar S (2007). MEGA4: Molecular evolutionary genetics analysis (MEGA) software version 40. Mol. Biol. Evol..

[107] Galli V (2015). Validation of reference genes for accurate normalization of gene expression for real time-quantitative PCR in strawberry fruits using different cultivars and osmotic stresses. Gene.

