# Supplementary material for: Genome-wide identification, and characterization of the CDPK gene family reveal their involvement in abiotic stress response in Fragaria x ananassa
Source: Sci Rep. 2020 Jul 6;10:11040. doi: 10.1038/s41598-020-67957-9 (PMC7338424; doi:10.1038/s41598-020-67957-9)

Genome-wide identification, and characterization of the CDPK gene family reveal their involvement in abiotic stress response in *Fragaria x ananassa*

Rosane Lopes Crizel^1^, Ellen Cristina Perin^2^, Isabel Lopes Vighi^3^, Rafael Woloski^3^, Amilton Seixas^3^, Luciano da Silva Pinto^3^, César Valmor Rombaldi^1^, Vanessa Galli^1,3*^

^1^Departamento de Ciência e Tecnologia Agroindustrial, Universidade Federal de Pelotas, Pelotas, Brasil

^2^Programa de Pós-Graduação em Tecnologia de Processos Químicos e Bioquímicos, Universidade Tecnologia Federal do Paraná, Pato Branco, Brasil

^3^Centro de Desenvolvimento Tecnológico, Universidade Federal de Pelotas, Pelotas, Brasil

*vane.galli@yahoo.com.br

## Supplementary Information: Alignment of the sequence of primers in sequences of strawberry (*Fragaria x ananassa*) CDPK


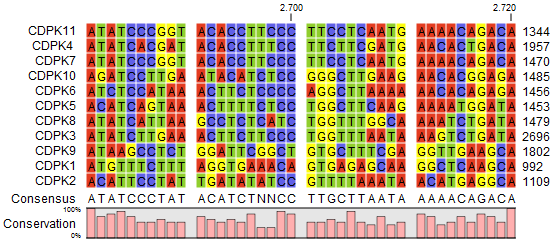


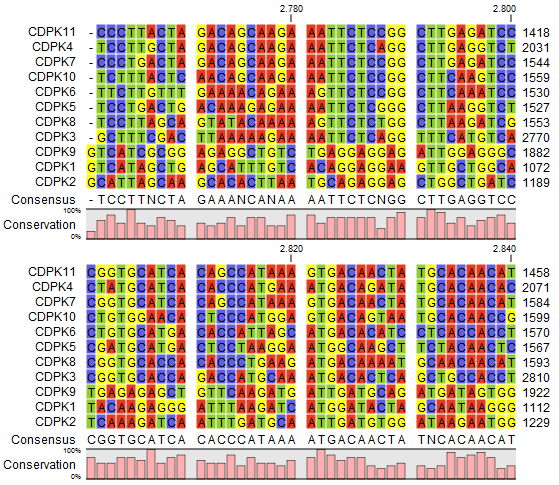


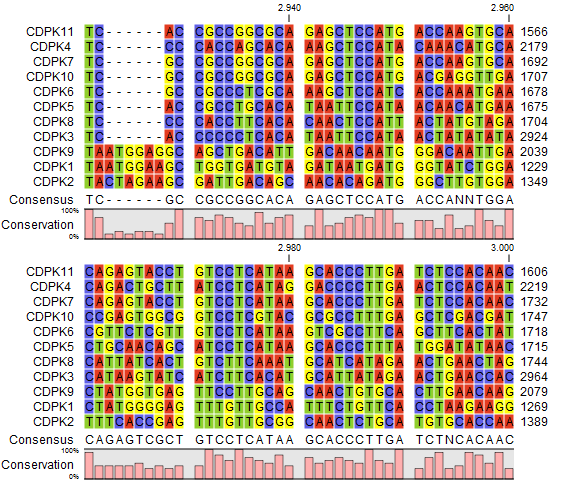


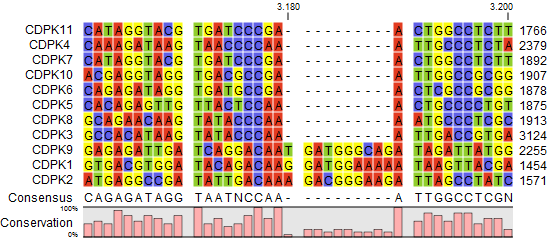


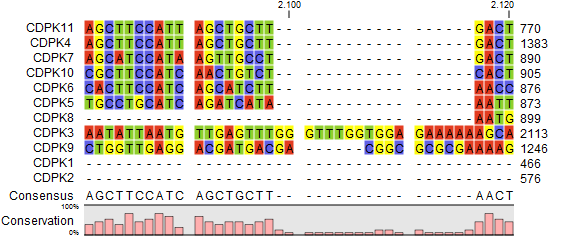


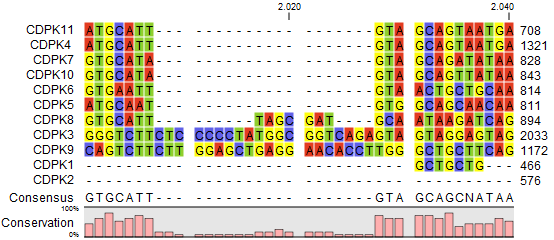


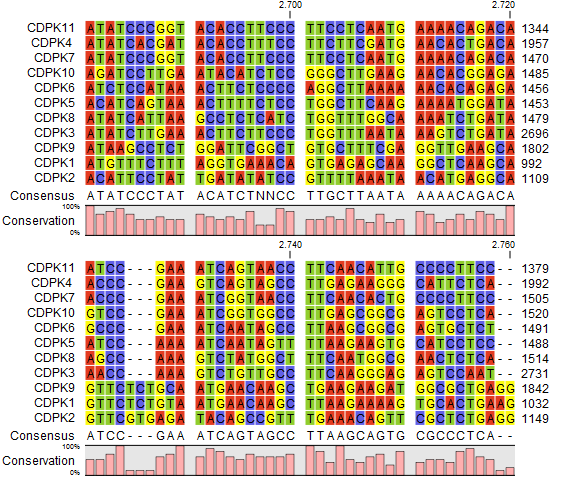


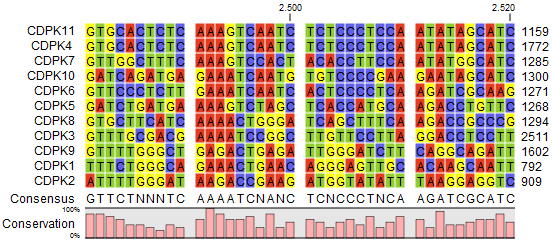


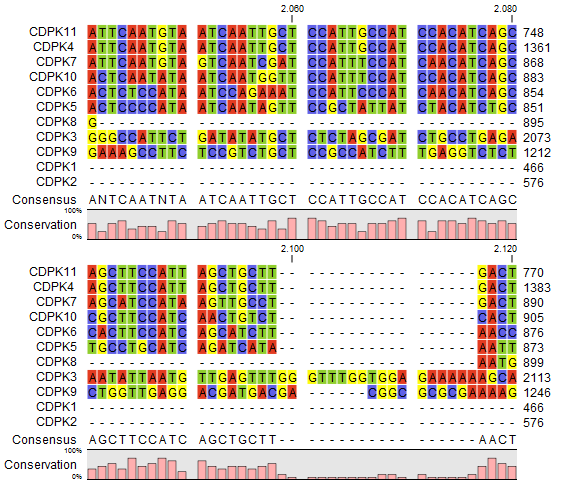


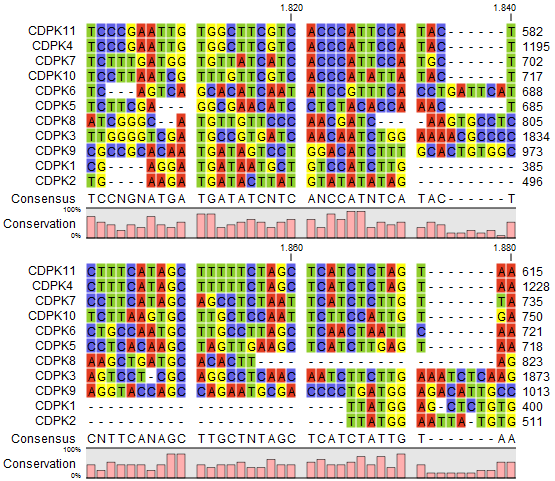


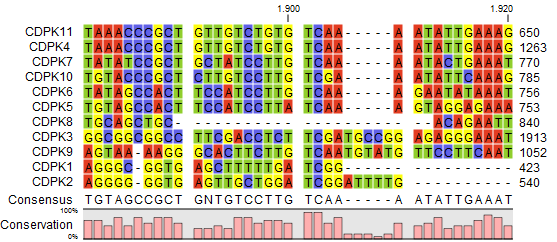


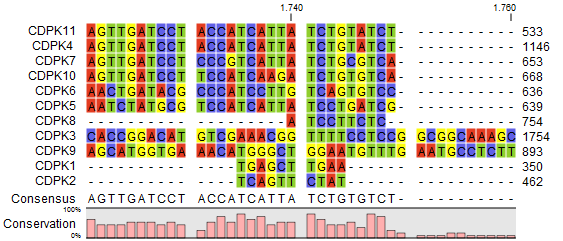


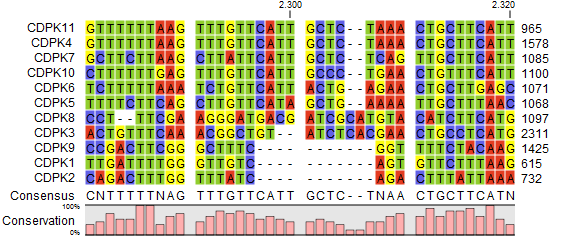


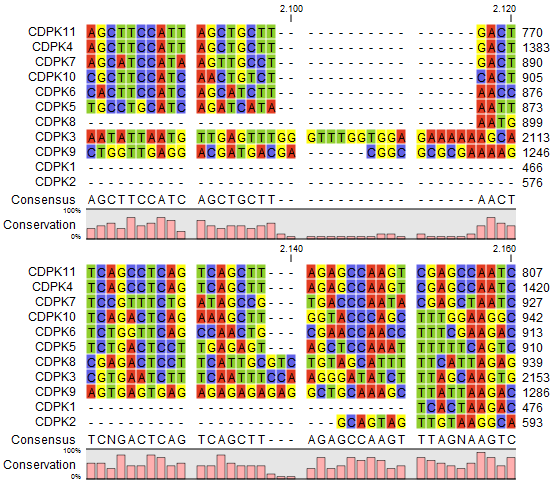


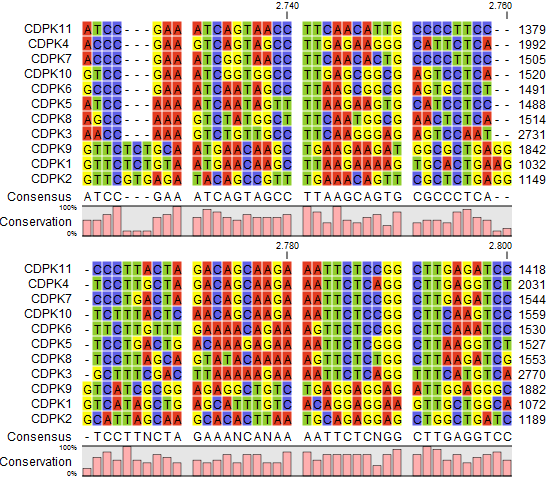


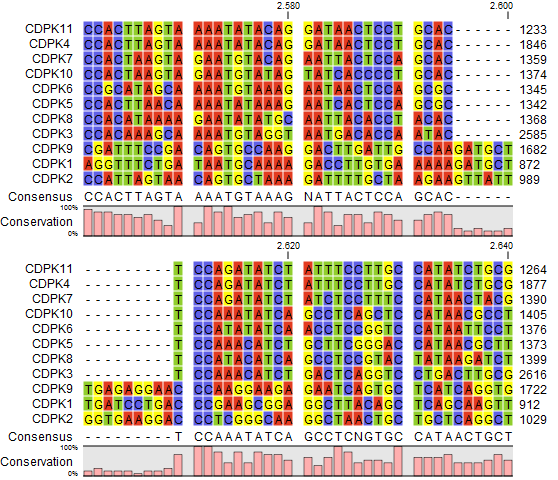


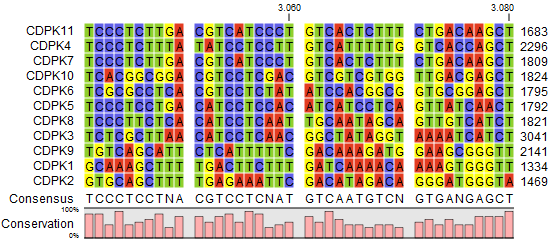


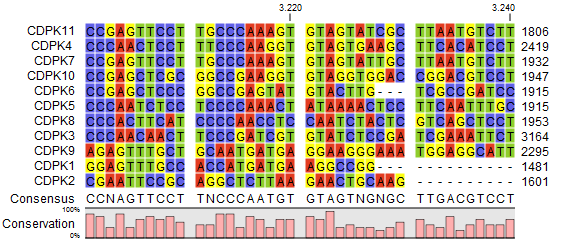


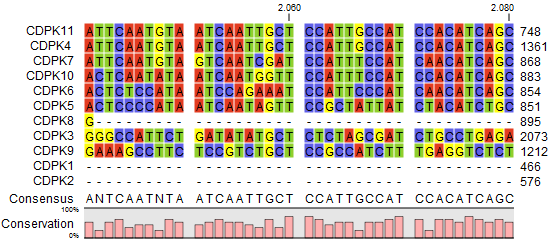


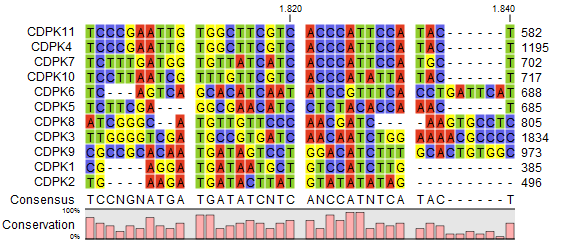


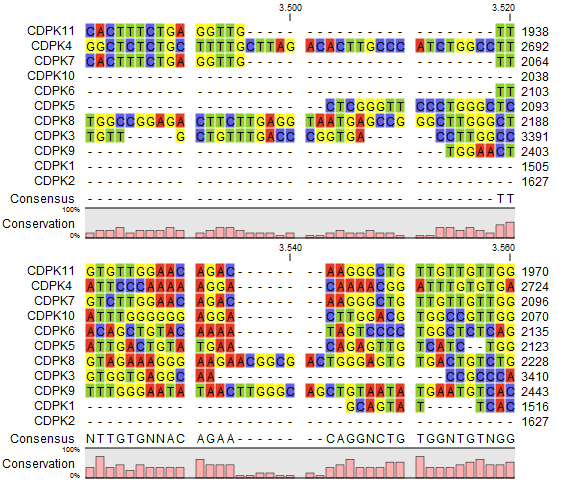


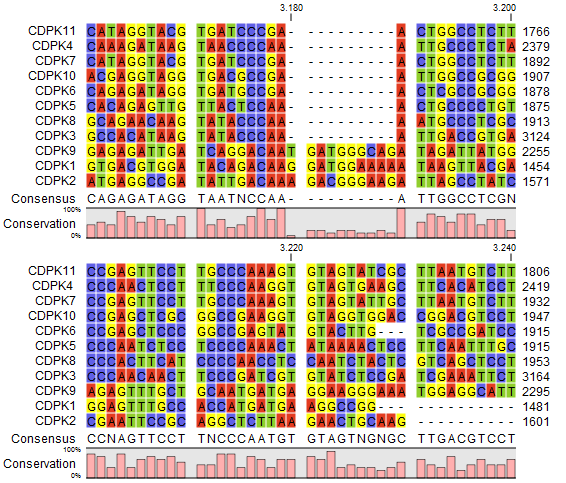

Supplement: Supplementary file 1 — Supplementary information [file 41598_2020_67957_MOESM1_ESM.docx]
