# Supplementary material for: Genome-wide identification, and characterization of the CDPK gene family reveal their involvement in abiotic stress response in Fragaria x ananassa
Source: Sci Rep. 2020 Jul 6;10:11040. doi: 10.1038/s41598-020-67957-9 (PMC7338424; doi:10.1038/s41598-020-67957-9)
Supplement: Supplementary file 2 — Supplementary information [file 41598_2020_67957_MOESM2_ESM.docx]

Genome-wide identification, and characterization of the CDPK gene family reveal their involvement in abiotic stress response in *Fragaria x ananassa*

Rosane Lopes Crizel^1^, Ellen Cristina Perin^2^, Isabel Lopes Vighi^3^, Rafael Woloski^3^, Amilton Seixas^3^, Luciano da Silva Pinto^3^, César Valmor Rombaldi^1^, Vanessa Galli^1,3*^

^1^Departamento de Ciência e Tecnologia Agroindustrial, Universidade Federal de Pelotas, Pelotas, Brasil

^2^Programa de Pós-Graduação em Tecnologia de Processos Químicos e Bioquímicos, Universidade Tecnologia Federal do Paraná, Pato Branco, Brasil

^3^Centro de Desenvolvimento Tecnológico, Universidade Federal de Pelotas, Pelotas, Brasil

*vane.galli@yahoo.com.br

**Supplementary Information**


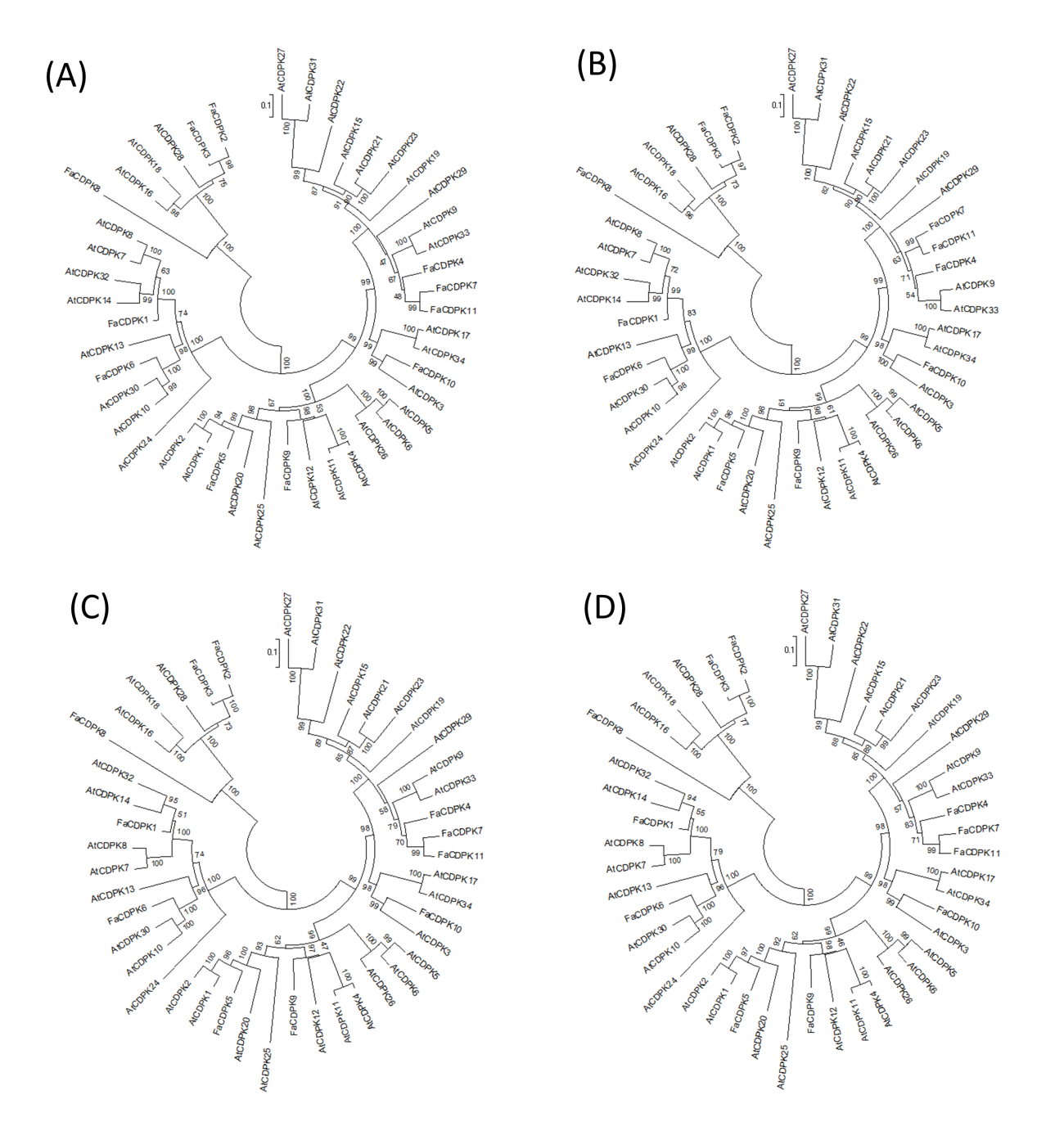


**Figure S1.**Phylogenetic relationship between the calcium dependent protein kinases (CDPK) of strawberry (*Fragraia x ananassa*) and Arabidopsis thaliana using different model substitutions. (A)Jones-Taylor-Thornton (JTT) model; (B) Dayhoff model; (C) Equal imput model; (D) Poisson model

**
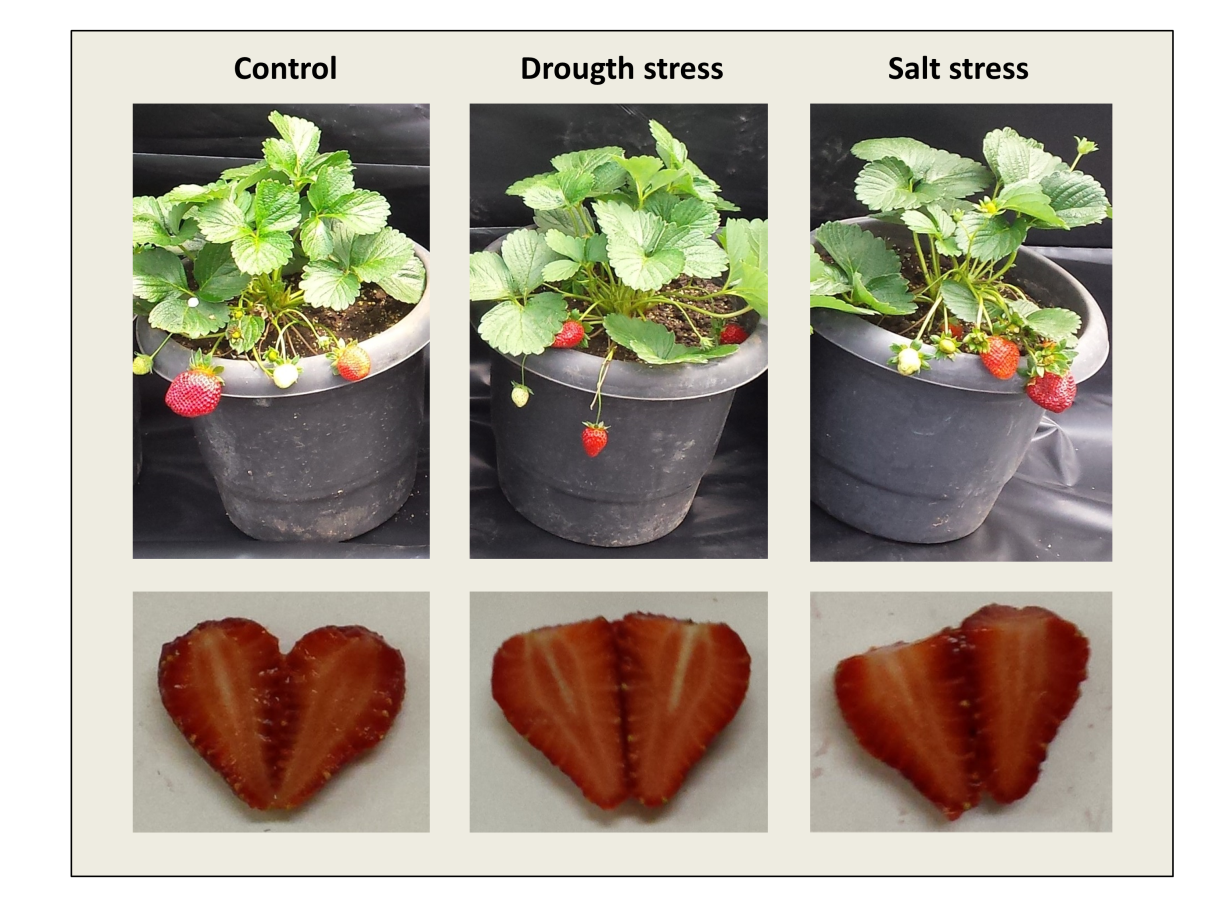
**

**Figure S2.** Strawberry plants subjected to different treatments (Control; Drougth stress; Salt stress). Source: the authors.

Table S1: Subcellular location and presence of transmembrane domains of calcium dependent kinases (CDPKs) identified in *F. ananassa*.

| **Gene name** | **Plant-PLoc**^a^ | **ChloroP**^b^ | **TMHMM**^c^ | **DeepLoc**^d^ |
| --- | --- | --- | --- | --- |
| FaCDPK1 | Chloroplast | - | + | Nucleus |
| FaCDPK2 | Chloroplast | Chloroplast (0.55) | - | Cell membrane |
| FaCDPK3 | Chloroplast | Chloroplast (0.50) | + | Cell membrane |
| FaCDPK4 | Chloroplast | Chloroplast (0.50) | + | Cell membrane |
| FaCDPK5 | Chloroplast | Chloroplast (0.56) | + | Peroxisome |
| FaCDPK6 | Chloroplast | - | + | Cell membrane |
| FaCDPK7 | Chloroplast | Chloroplast (0.53) | + | Cell membrane |
| FaCDPK8 | Chloroplast | Chloroplast (0.56) | + | Cell membrane |
| FaCDPK9 | Chloroplast | Chloroplast (0.51) | - | Cytoplasm |
| FaCDPK10 | Chloroplast | Chloroplast (0.52) | - | Cell membrane |
| FaCDPK11 | Chloroplast | Chloroplast (0.55) | + | Cell membrane |

^a^Localization prediction by Plant-PLoc (http://www.csbio.sjtu.edu.cn/bioinf/plant/)

^b^Localization prediction by ChloroPv 1.1 (http://www.cbs.dtu.dk/services/ChloroP/)

^c^ Localization prediction by TMHMM v. 2.0 (http://www.cbs.dtu.dk/services/TMHMM/)

^d^Localization prediction by DeepLoc v. 1.0 (http://www.cbs.dtu.dk/services/DeepLoc/)

Table S2. Synteny blocks of CPK genes between *Fragaria x ananassa* and Arabidopsis.

| **gene name** | | **Ka** | **Ks** | **Ka/Ks** | **Types of selection** | **Time (MYA)** |
| --- | --- | --- | --- | --- | --- | --- |
| **gene1** | **gene2** |  |  |  |  |  |
| FaCDPK1 | AtCDPK32 | 0.08 | 1.81 | 0.04 | Purifying | 60.2 |
| FaCDPK2 | FaCDPK3 | 0.03 | 0.07 | 0.47 | Positive | 2.4 |
| FaCDPK2 | AtCDPK16 | 0.15 | 6.13 | 0.02 | Purifying | 204.3 |
| FaCDPK2 | AtCDPK18 | 0.16 | 2.34 | 0.07 | Positive | 78.1 |
| FaCDPK3 | AtCDPK16 | 0.18 | 7.53 | 0.02 | Positive | 250.9 |
| FaCDPK3 | AtCDPK18 | 0.16 | 3.73 | 0.04 | Positive | 124.2 |
| FaCDPK4 | FaCDPK11 | 0.11 | 0.58 | 0.18 | Purifying | 19.3 |
| FaCDPK4 | FaCDPK7 | 0.16 | 2.01 | 0.08 | Positive | 66.9 |
| FaCDPK4 | AtCDPK33 | 0.14 | 3.80 | 0.04 | Purifying | 126.5 |
| FaCDPK4 | AtCDPK9 | 0.14 | 2.43 | 0.06 | Purifying | 81.0 |
| FaCDPK5 | AtCDPK1 | 0.11 | 2.99 | 0.04 | Positive | 99.7 |
| FaCDPK5 | AtCDPK5 | 0.11 | 2.99 | 0.04 | Purifying | 99.7 |
| FaCDPK6 | AtCDPK10 | 0.10 | 3.32 | 0.03 | Positive | 110.7 |
| FaCDPK6 | AtCDPK30 | 0.10 | 2.88 | 0.04 | Positive | 95.9 |
| FaCDPK7 | FaCDPK11 | 0.04 | 0.45 | 0.11 | Positive | 14.9 |
| FaCDPK7 | AtCDPK33 | 0.18 | 5.45 | 0.03 | Purifying | 181.8 |
| FaCDPK7 | AtCDPK9 | 0.20 | 3.07 | 0.07 | Purifying | 102.2 |
| FaCDPK9 | AtCDPK4 | 0.17 | 8.43 | 0.02 | Purifying | 280.9 |
| FaCDPK9 | AtCDPK11 | 0.16 | 4.98 | 0.03 | Purifying | 165.9 |
| FaCDPK10 | AtCDPK3 | 0.15 | 4.27 | 0.04 | Positive | 142.3 |
| FaCDPK11 | AtCDPK33 | 0.18 | 5.34 | 0.03 | Purifying | 177.9 |
| FaCDPK11 | AtCDPK9 | 0.19 | 3.35 | 0.06 | Purifying | 111.7 |
